# NF-κB signaling as a critical inflammatory node in pulmonary arterial hypertension: from vascular remodeling to right heart failure

**DOI:** 10.3389/fimmu.2026.1921908

**Published:** 2026-09-01

**Authors:** Yan He, Yingying Zhu, Qihong Lv, Hongxia Wu

**Affiliations:** Department of Respiratory and Critical Care Medicine, West China Hospital, Sichuan University, Chengdu, Sichuan, China

**Keywords:** EndMT, epitranscriptomic, microRNAs, NF-κB, PAH

## Abstract

Pulmonary arterial hypertension (PAH) is a progressive disease characterized by pulmonary vascular remodeling, leading to hemodynamic impairment and right heart dysfunction, and ultimately right heart failure and death. Despite advances in understanding its pathogenesis, PAH remains associated with poor prognosis and high mortality compared with other respiratory diseases, posing a significant global health burden. Current therapies primarily target the endothelin, nitric oxide, and prostacyclin pathways to achieve vasodilation and symptomatic relief; however, they have limited efficacy in reversing established vascular remodeling. The nuclear factor-κB (NF-κB) signaling pathway, a central regulator of inflammation and immune responses, plays a critical role in pulmonary vascular remodeling and endothelial-to-mesenchymal transition (EndMT). Targeting NF-κB has therefore emerged as a promising therapeutic strategy for PAH. Nevertheless, controversies remain regarding its mechanisms, therapeutic efficacy, and potential risks, particularly in combination strategies. This review summarizes current understanding of PAH pathogenesis and recent advances in targeting the NF-κB pathway.

## 1 Introduction

PAH is a syndrome characterized by pulmonary vascular remodeling and elevated pulmonary vascular resistance (1). It is classified into five groups based on etiology, pathophysiology, and clinical features, including idiopathic/heritable PAH, left heart disease–associated PAH, lung disease/hypoxia-associated PH, chronic thromboembolic pulmonary hypertension, and multifactorial/unclear mechanisms. According to the updated pulmonary arterial hypertension standards from the 6th World Symposium on PAH (2018), in terms of hemodynamics, the definition of PAH is that the mean pulmonary artery pressure (mPAP) is greater than 20 mmHg, the pulmonary artery wedge pressure is less than or equal to 15 mmHg, and the pulmonary vascular resistance (PVR) is greater than 2 Wood units (2, 3). Pulmonary vascular lesions in PAH are characterized by vasoconstriction and structural remodeling, leading to progressive dyspnea, exercise intolerance, right heart failure, and death (2, 4). A French registry reported an annual incidence of ~2.4 per 100,000 person-years and adult prevalence of 15 per 100,000; the US REVEAL registry reported 2.0 per 100,000 and 10.6 per 100,000, respectively (5–7). The latest data from a US registry shows that the one-year, three-year and five-year mortality rates for PAH are 15%, 32% and 43% respectively, posing a challenge to the global public health system (7–9). Currently, the pathogenesis of PAH is unclear, and the exact pathophysiological mechanism remains controversial (10). Over the past three decades, the treatment direction of PAH has mainly focused on endothelin receptors, nitric oxide, and prostacyclin pathways, which have significantly improved pulmonary hemodynamic and exercise capacity in PAH and reduced clinical deterioration events, but the median survival period and prognosis have not significantly improved (11). Sotatercept, a first-in-class activin receptor type IIA-Fc (ActRIIA-Fc) fusion protein that traps activin and other TGF-β superfamily ligands, was approved by the FDA in March 2024 as a disease-modifying therapy targeting vascular remodeling. The phase 3 STELLAR trial demonstrated that adding sotatercept to background therapy improved 6-minute walk distance by ~40 meters versus placebo (12). A pooled analysis of PULSAR and STELLAR confirmed improvements in PVR, WHO functional class, and right ventricular structure and function. The phase 3 HYPERION trial met its primary endpoint in newly diagnosed patients, while the ZENITH trial was stopped early due to a 76% reduction in the composite endpoint of all-cause death, transplantation, or PAH-related hospitalization (HR: 0.24) (12). Interim SOTERIA data support long-term durability of clinical benefits (13). Meta-analysis of 601 patients confirmed significant improvements across multiple endpoints (14–17). These landmark clinical successes underscore a paradigm shift in PAH therapeutics—moving beyond symptom relief toward directly counteracting the underlying vascular pathology. The unprecedented efficacy of sotatercept, which mechanistically restores the balance between pro-proliferative (activin/ActRIIA) and anti-proliferative (BMPR2, bone morphogenetic protein receptor type 2) signaling, reinforces the notion that targeting core pathogenic pathways can yield substantial clinical benefit. This paradigm shift highlights an urgent need to further elucidate the molecular networks driving pulmonary vascular remodeling. A detailed understanding of PAH-related pathogenic molecular mechanisms is therefore vital for exploring novel targeted treatments, improving prognosis, and increasing overall survival (13).

PAH is driven by multiple pathological processes, including inflammation, apoptosis resistance, thrombosis, excessive proliferation, endothelial-to-mesenchymal transition (EndMT), epigenetic dysregulation, and dysfunction of pulmonary arterial endothelial and smooth muscle cells (18). Multiple signaling pathways regulate these processes and participate in PAH promotion and progression. Nuclear factor kappa-B (NF-κB) is a transcription factor recently found to play an important regulatory role in various cardiopulmonary diseases, including PAH, and orchestrates in inflammation and immune responses (19–21). The activation of NF-κB proteins is strictly regulated. Classical pathway activation requires release of p65/p50 subunits from the inhibitor κB complex and translocation of the p65/p50 heterodimer to the nucleus, activating target genes involved in proliferation, differentiation, inflammation, and angiogenesis (22, 23). Inappropriate NF-κB activation leads to autoimmune disorders, chronic inflammation, and malignant tumor progression. Studies show that abnormal NF-κB activation is closely related to PASMC proliferation and enhanced vascular remodeling, and simultaneously exerts pro-PAH effects by aberrantly regulating MicroRNA expression, causing irreversible pulmonary vascular changes. This suggests that NF-κB pathway participates in PAH development through multiple pathological mechanisms, resulting in reduced exercise capacity and ongoing clinical decline. This paper will systematically summarize the activation mechanisms of classical and non-classical NF-κB signaling pathways in PAH, with emphasis on their regulation. In addition, it will discuss the regulatory role of NF-κB in pulmonary vascular remodeling, endothelial dysfunction, EndMT, RV decompensation, and epigenetics, and evaluate therapeutic approaches targeting the NF-κB-related signaling network and their potential for clinical translation.

## 2 NF-κB pathway

### 2.1 Classic NF-κB signaling pathway

NF-κB is a highly conserved family of inducible transcription factors that regulates immunity, inflammation, cell survival, proliferation, and stress responses. RelA (p65), RelB, c-Rel, NF-κB1 (p50/p105), and NF-κB2 (p52/p100) are the five members of the NF-κB family in mammals (20, 24, 25). All members contain an N-terminal Rel homology domain (RHD) responsible for DNA binding, dimerization, and interaction with IκB proteins.NF-κB proteins are divided into transcriptionally active members (RelA, RelB, and c-Rel) and precursor proteins (p50 and p52), which regulate gene transcription depending on dimer composition (26). Under resting conditions, NF-κB dimers are retained in the cytoplasm by inhibitory IκB proteins (IκBα, IκBβ, and IκBϵ) (27, 28). When cells are stimulated, the i-κB protein undergoes phosphorylation-dependent ubiquitination and proteasomal degradation, releasing the NF-κB dimer into the nucleus and activating the transcription of target genes. Aberrant activation contributes to chronic inflammation, autoimmune diseases, and tumor progression (27, 29). In PAH, the canonical NF-κB pathway is the predominant inflammatory signaling cascade. It is activated by pro-inflammatory stimuli including tumor necrosis factor-α (TNF-α), interleukin-1β (IL-1β), lipopolysaccharide (LPS), oxidative stress, and hypoxia.

### 2.2 Non-classical NF-κB signaling pathway

The non-canonical NF-κB pathway induces a slower but more sustained signaling response compared with the canonical pathway. It is selectively activated by members of the tumor necrosis factor receptor superfamily, including CD40, receptor activator of NF-κB (RANK), lymphotoxin β receptor (LTβR), and B-cell activating factor receptor (BAFFR) (30). This pathway depends primarily on NF-κB-inducing kinase (NIK) and IKKα, rather than IKKβ/NEMO. Under basal conditions, NIK is constitutively degraded via TRAF2/TRAF3/cIAP-mediated ubiquitination. Upon receptor stimulation, TRAF3 degradation stabilizes NIK, leading to its accumulation and activation of IKKα homodimers. Activated IKKα phosphorylates NF-κB precursor p100, promoting its partial proteasomal processing into p52 (31).The resulting RelB/p52 heterodimer translocates to the nucleus and regulates genes involved in immune homeostasis, chronic inflammation, lymphoid organogenesis, and cell survival (32).

Compared with the canonical pathway, the biological significance of non-canonical NF-κB signaling in PAH remains largely unexplored. However, several characteristics suggest that this pathway may be particularly relevant to the chronic and progressive nature of pulmonary vascular remodeling. Unlike acute inflammatory activation mediated predominantly by canonical NF-κB signaling, the non-canonical pathway is specialized in regulating persistent immune responses and lymphocyte activation (33). These functions closely overlap with key pathological features of PAH, including sustained perivascular inflammation, immune cell accumulation, adventitial remodeling, and progressive vascular fibrosis.

Emerging evidence from cardiovascular and pulmonary inflammatory diseases suggests that non-canonical NF-κB signaling may regulate vascular remodeling through multiple mechanisms (34). Activation of LTβR/NIK/IKKα signaling has been implicated in chronic vascular inflammation and endothelial activation (35), whereas dysregulated RelB/p52 activity may influence macrophage polarization, fibroblast activation, and extracellular matrix remodeling (36, 37). Given that immune cells, pulmonary endothelial cells, smooth muscle cells, and adventitial fibroblasts all participate in PAH progression, the non-canonical NF-κB pathway may represent an additional regulatory layer linking immune dysregulation with structural vascular changes.

Critically, several significant questions remain unanswered regarding its involvement in PAH. First, the specific upstream stimuli responsible for non-canonical NF-κB activation in PAH are unknown (33). Whether hypoxia, mechanical stress, inflammatory cytokines, or disease-associated molecular patterns activate this pathway requires further investigation. Second, the cell-specific functions of non-canonical NF-κB signaling within pulmonary vascular compartments remain unclear. Although canonical NF-κB activation has been extensively demonstrated in pulmonary arterial endothelial cells and PASMCs, direct evidence regarding RelB/p52 signaling in these cell populations is limited (38). Third, the interaction between canonical and non-canonical NF-κB pathways during PAH progression remains poorly defined. It is possible that early canonical activation initiates inflammatory responses, whereas sustained non-canonical activation maintains chronic inflammation and remodeling (31). Finally, whether selective modulation of the non-canonical pathway can provide therapeutic benefits while avoiding systemic immunosuppression remains an critical translational challenge (39).

Although direct evidence linking non-canonical NF-κB activation to PAH remains limited, studies in vascular inflammation provide important mechanistic clues. LTβR-mediated activation of NIK/IKKα-dependent NF-κB signaling has been shown to induce RelB/p52-driven inflammatory gene expression in endothelial cells, suggesting that non-canonical NF-κB signaling may participate in sustained vascular inflammation and endothelial activation (40). Unlike canonical NF-κB, which predominantly mediates rapid inflammatory responses, the non-canonical pathway generates prolonged transcriptional programs involved in immune regulation and tissue remodeling (33). Therefore, non-canonical NF-κB signaling may represent an unexplored regulatory layer connecting chronic immune activation with progressive pulmonary vascular remodeling in PAH. The activation kinetics, upstream stimuli, and downstream functions of the canonical and non-canonical pathways differ markedly. To facilitate direct comparison, the key features of both pathways are summarized in **Table 1**.

**Table 1:** Comparison of canonical and non-canonical NF-κB signaling pathways.

Feature	Canonical NF-κB pathway	Non-canonical NF-κB pathway
Upstream stimuli	TNF-α, IL-1β, LPS, oxidative stress	CD40L, LTβR, BAFF, RANKL
Key kinases	IKKα, IKKβ, NEMO (IKKγ)	NIK, IKKα (homodimers)
IκB proteins involved	IκBα, IκBβ, IκBϵ	p100 (acts as IκB for RelB)
NF-κB dimer activated	p65/p50 (RelA/p50)	RelB/p52
Kinetics	Rapid (minutes)	Slow (hours)
Primary function	Acute inflammation, cell survival, proliferation	Chronic inflammation, lymphoid organogenesis, B-cell survival
Therapeutic targeting	IKKβ inhibitors (IMD-0354), upstream modulators	NIK inhibitors, IKKα inhibitors (under development)
Relevance to PAH	Well established	Emerging, less defined

### 2.3 Functional significance of NF-κB activation in PAH

The canonical and non-canonical NF-κB pathways form an integrated regulatory network driving inflammation and vascular remodeling in PAH. The canonical NF-κB pathway primarily mediates acute inflammatory responses, oxidative stress, endothelial dysfunction, and pulmonary arterial smooth muscle cell (PASMC) proliferation. In contrast, the non-canonical pathway is more closely associated with chronic immune activation and persistent vascular remodeling. Notably, NF-κB functions as a central signaling hub in PAH. It coordinates multiple pathological processes, including pulmonary vascular remodeling, endothelial plasticity, and right ventricular (RV) maladaptation (41). This coordination is achieved through crosstalk with various molecular partners, such as hypoxia-inducible factor-1α (HIF-1α), STAT3, BMPR2, reactive oxygen species (ROS), IL-6, and metabolic pathways (42). Additionally, NF-κB is involved in epigenetic regulation. Collectively, the NF-κB-centered signaling network plays a pivotal role in both the initiation and progression of PAH. Therefore, it represents a potential therapeutic target for disease modification. Given the central role of NF-κB in PAH, multiple intervention strategies have been tested in experimental models. Based on target maturity and mechanism of action, representative approaches are classified into direct inhibitors, upstream modulators, and indirect regulators in **Tables 2A** and **2B**.

**Table 2A:** Direct catalytic inhibitors of the NF-κB pathway in PAH.

Interventions	Molecular target/Action mechanism	Action pathway	Effects	Combination	Experimental model	
Direct NF-κB Inhibitors						
IMD-0354	IKKβ inhibitor (ATP-competitive)	Inhibition of Erk 1/2/NF-kB/MCP-1pathway	a. Inhibit the progression of PAH b. Inhibit pulmonary vascular remodeling a. Inhibit the proliferation and migration of PASMCs	Monotherapy	a. Monocrotaline-induced pulmonary hypertension mouse model	
BET inhibitor JQ1	BRD4 (bromodomain-containing protein 4)	Inhibition of NF-κB/IL-6 pathway	a. Inhibit the progression of PAH	Monotherapy	a. Peripheral lung tissue	
Secin H3	NF-κB signaling inhibitor (mechanism under investigation)	Inhibition of NF-κB pathway	a. Inhibit the progression of PAH b. Restore the expression of BMPR2	Monotherapy	a. Pulmonary artery endothelial cells b. Hypoxia-induced pulmonary hypertension mouse model c. Monocrotaline-induced pulmonary hypertension mouse model	

**Table 2B:** Indirect NF-κB modulators in PAH: upstream pathway blockade, homeostatic regulation, and transcriptional crosstalk.

Interventions	Primary Target	Action pathway	Mechanism of Indirect Regulation	Combination	Experimental Model
Upstream Pathway Modulators					
Resveratrol	SphK1 inhibitor	Inhibition of SphK1/S1P/NF-κB/cyclin D1 pathway	Inhibits SphK1 → reduces S1P-mediated IKK activation → decreases NF-κB nuclear translocation and cyclin D1 transcription	Monotherapy	a. Pulmonary artery endothelial cells b. Hypoxia-induced pulmonary hypertension mouse model
Cinnamaldehyde	TLR4 signaling inhibitor	Inhibition of TLR4/NF-kB/HIF-1a pathway	Antagonizes TLR4 → blocks MyD88/TRIF signaling → prevents IκBα phosphorylation/degradation and suppresses HIF-1α activity	Monotherapy	a. Hypoxia-induced pulmonary hypertension mouse model b. Su/Hx-induced pulmonary hypertension mouse model
Melatonin (MLT)	TLR4/NF-κB pathway inhibitor pathway	Inhibition of TLR4/NF-kβ pathway	Suppresses TLR4 signaling and modulates redox-sensitive IKK phosphorylation → reduces NF-κB activation and downstream IL-6/TNF-α production	Monotherapy	a. Monocrotaline-induced pulmonary hypertension mouse model
Sevoflurane	TRAF6/NF-κB pathway inhibitor	Inhibition of TRAF6/NF-κB pathway	Inhibits TRAF6 → prevents IKK complex ubiquitination and phosphorylation → limits inflammatory amplification and PASMC proliferation	Inhalation anesthetic	a. Monocrotaline-induced pulmonary hypertension mouse model
Thymoquinone (TQ)	p38 MAPK inhibitor	Inhibition of p38 MAPK/NF-κB pathway	Inhibits p38 MAPK → reduces MAPK-IKK crosstalk and IκBα degradation → diminishes NF-κB nuclear translocation	Monotherapy	a. Monocrotaline-induced pulmonary hypertension mouse model
Indirect NF-κB Modulators					
Dimethyl fumarate (DMF)	Nrf2 activator	Inhibition of NF-κB pathway	Activates Nrf2 → induces antioxidant genes (HO-1, NQO1) → reduces ROS-mediated IKK activation; directly modifies p65 cysteine residues;	Monotherapy	a. pulmonary artery smooth muscle cells b. Hypoxia-induced pulmonary hypertension mouse model
Zinc finger protein A20 (overexpression)	TNFAIP3 (A20)	Inhibition of NF-κB pathway	Removes K63-linked ubiquitin chains from RIP1, TRAF6, and IKKγ (NEMO), blocking upstream activation signals	Monotherapy	a. Hypoxia-induced pulmonary hypertension mouse model
Corosolic acid	NF-κB/STAT3 dual pathway inhibitor	Inhibition of NF-κB/STAT3 pathway	Reduces STAT3 phosphorylation → decreases NF-κB transcriptional activity; inhibits PASMC proliferation	Monotherapy	a. pulmonary artery smooth muscle cells b. Monocrotaline-induced pulmonary hypertension mouse model
Baicalein	AKT/ERK pathway inhibitor	Inhibition of AKT/ERK/NF-κB pathway	Inhibits AKT and ERK1/2 phosphorylation → decreases IKK activity and IκBα degradation	Monotherapy	a. Monocrotaline-induced pulmonary hypertension mouse model

## 3 The regulatory network of NF-κB on pulmonary vascular remodeling in PAH

### 3.1 Cell-type-specific roles of NF-κB in pulmonary vascular remodeling

NF-κB activation in PAECs primarily contributes to endothelial inflammatory activation, barrier dysfunction, aberrant angiogenesis, and EndMT, while its effects on apoptosis are context-dependent. In human PAH lung tissues, increased nuclear localization of NF-κB subunits has been detected in vascular lesions, although cell-type-specific attribution remains technically challenging. SOX17(SRY-box transcription factor 17) silencing in HPAECs leads to excessive NF-κB p65 activity, markedly elevating CXCL10 and CXCL11 chemokine release and promoting lymphocyte chemotaxis, implicating endothelial NF-κB in immune cell recruitment in SOX17-associated PAH (43). Targeted endothelial FTO (fat mass and obesity-associated protein, an N6-methyladenosine demethylas) knockout reduces hypoxia-induced PAH progression partially through attenuation of TNF-α/NF-κB-associated inflammatory signaling, and decreasing neutrophil infiltration, highlighting the pathogenic role of endothelial NF-κB in inflammatory cell recruitment (44). Experimental studies suggest that endothelial ALDH2 deficiency promotes vascular remodeling through activation of the p38/NF-κB/IL-6 axis. TNF-induced endothelial NF-κB activation can alter TGF-β/BMP pathway balance by promoting SMAD2/3 activation and impairing endothelial BMP homeostasis. Notably, experimental activation of NF-κB in dendritic cells has been shown to promote IL-6-dependent pulmonary vascular remodeling (45).

Aberrant proliferation and migration of PASMCs are central to pulmonary vascular remodeling in PAH, with NF-κB signaling playing a critical role in driving these pathological processes through multiple convergent pathways. Nucleotide-binding oligomerization domain-like receptor C3 (NLRC3) is significantly downregulated in lung tissues from patients with high-altitude pulmonary hypertension (HAPH) and in hypoxia-induced PH mice. NLRC3 downregulation activates the IKK/NF-κB p65/HIF-1α signaling axis, driving hypoxia-induced PASMC proliferation and migration (42). Additionally, nicotinamide phosphoribosytransferase (NAMPT) promotes platelet-derived growth factor (PDGF)-induced PASMC proliferation by activating TLR4/NF-κB-mediated upregulation of polo-like kinase 4 (PLK4) (46). The histone demethylase JARID1B (Jumonji AT-rich interactive domain 1B) is upregulated in PASMCs of mice with chronic hypoxia-induced PH and binds directly to the NF-κB promoter to enhance its expression, leading to vascular endothelial growth factor A (VEGF-A) upregulation that promotes PASMC proliferation and migration (47). Furthermore, NF-κB p65 signaling directly regulates the microRNA-17 (miR-17) promoter, increasing miR-17 expression in PASMCs. This process inhibits retinoblastoma (RB) protein function, stimulates PASMC proliferation, promotes G1/S phase transition, and upregulates proliferating cell nuclear antigen (PCNA) and E2F1 expression (48).

Crosstalk pathways converging on NF-κB involve Akt/mTORC1, RISP, and MAL signaling, which enhance NF-κB transcriptional activity and promote vascular remodeling. Hypoxia activates the Akt/mammalian target of rapamycin complex 1 (mTORC1) signaling pathway, which induces abnormal vascular injury responses. mTORC1 phosphorylates IKKα/β, mediating crosstalk between the Akt/mTORC1 and NF-κB pathways and enhancing NF-κB transcriptional activity. This results in upregulated dipeptidyl peptidase-4 (DPP4) expression in PASMCs and promotes vascular remodeling (49). The Rieske iron-sulfur protein (RISP) stimulates PASMC proliferation and pulmonary arterial remodeling via the NF-κB/cyclin D1 signaling pathway, inducing dissociation of the FK506 binding protein 12.6/ryanodine receptor 2 (FKBP12.6/RyR2) complex and contributing to sustained PH (50). Moreover, hypoxia upregulates myelin and lymphocyte protein (MAL), which activates the NF-κB pathway and promotes excessive proliferation and anti-apoptosis in human PASMCs *in vitro (*51).Numerous experimental models have confirmed that the inhibition of NF-κB can reduce the proliferation of PASMC. Zinc finger protein A20, a negative feedback regulator of NF-κB activity, exerts a protective effect by downregulating NF-κB activity, thereby inhibiting excessive PASMC proliferation and pulmonary vascular remodeling (52). Similarly, thymoquinone attenuates monocrotaline-induced pulmonary artery remodeling and PASMC proliferation through inhibition of p38 MAPK and NF-κB activation (53). Furthermore, in lupus-associated PAH, high mobility group box 1 (HMGB1) suppresses hypoxia-induced PASMC proliferation, migration, and collagen synthesis via the TLR4/NF-κB pathway, highlighting the role of NF-κB in autoimmune forms of PAH. Collectively, the IKK/NF-κB p65/HIF-1α, TLR4/NF-κB/PLK4, NF-κB/miR-17/RB, Akt/mTORC1/NF-κB/DPP4, RISP/NF-κB/cyclin D1, and MAL/NF-κB axes converge to drive PASMC proliferation, migration, and resistance to apoptosis, contributing significantly to vascular remodeling in PAH. Protective molecules such as A20 attenuate these effects by suppressing NF-κB activity, underscoring the therapeutic potential of targeting NF-κB signaling in PASMCs.

In macrophages, NF-κB regulates polarization toward pro-inflammatory (M1) or pro-remodeling (M2a) phenotypes (41). In a severe PAH model induced by left pneumonectomy plus monocrotaline, pulmonary interstitial macrophages initially exhibited a predominant M1-polarized phenotype in the early stage, which transitioned to an M2a-polarized phenotype in the later stage and eventually stabilized (54). M2a macrophages play a crucial role in PAH progression by promoting PASMC proliferation, and low-dose paclitaxel alleviates severe PAH by suppressing M2a polarization via NF-κB inhibition. NF-κB activation in macrophages drives the production of IL-1β, IL-6, TNF-α, and MCP-1/CCL2, which in turn promote immune-cell recruitment, amplify perivascular inflammation, and establish a self-sustaining inflammatory microenvironment. NLRX1 deficiency upregulates inflammatory mediators by activating NF-κB, while NLRX1 may suppress hypoxia-induced lung inflammation and alleviate pulmonary hypertension through its anti-inflammatory effects (55).

In adventitial fibroblasts, NF-κB activation promotes extracellular matrix deposition, myofibroblast differentiation, and chemokine production (56). TNF-Tg model studies demonstrate that NF-κB signaling pathway, JAK/STAT signaling pathway, and interferon-mediated inflammation drive changes in fibroblast subpopulations, including increases in Col14^+^ fibroblasts and reductions in Col13^+^ fibroblasts (45). Activated adventitial fibroblasts play an important role in regulating vasa vasorum growth, which contributes to ongoing vascular remodeling by acting as a conduit for delivery of inflammatory and progenitor cells (57). Intracellular complement system activation drives metabolic and proinflammatory reprogramming of vascular fibroblasts in pulmonary hypertension, further amplifying the fibrotic response (58).

**Figure 1:**
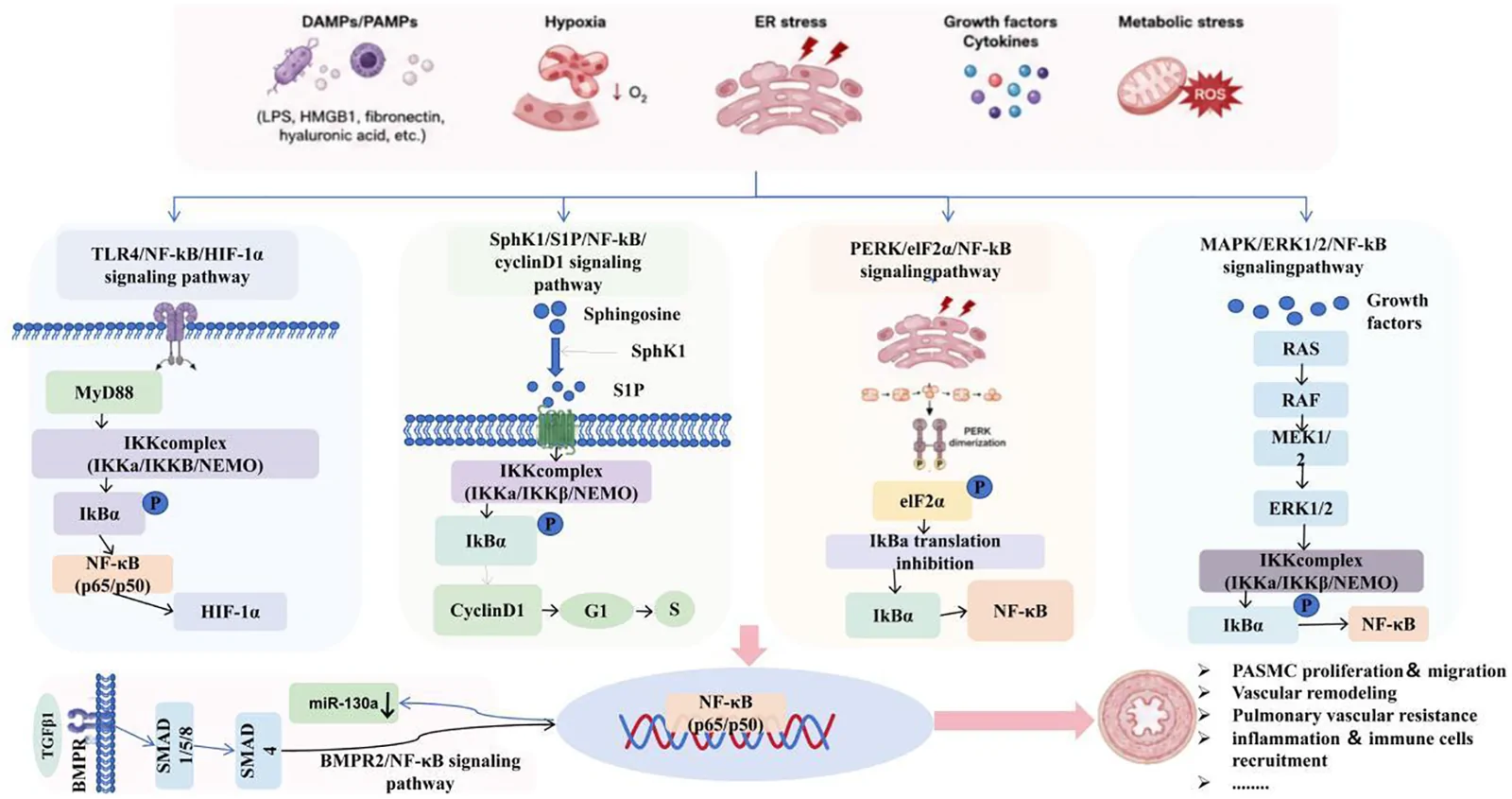
NF-κB as a central signaling hub integrating pulmonary vascular remodeling in PAH.

In pericytes, TNF-Tg model single-cell RNA sequencing revealed reductions in pericyte populations, with pathway analysis demonstrating NF-κB-mediated inflammation contributing to pericyte dysfunction and altered cell-cell communication. TNIP2 and TRAF2 variants have been identified as NF-κB regulators in pericytes, promoting pericyte proliferation and contributing to PH pathogenesis (59). These findings suggest that NF-κB signaling in pericytes may represent an underappreciated mechanism in pulmonary vascular remodeling. How do diverse pathological stimuli converge on NF-κB and trigger pulmonary vascular remodeling? **Figure 1** provides a global network view, integrating upstream signals, the central IKK/NF-κB node, and downstream effector responses, offering a conceptual framework for the detailed pathway discussions that follow.

This schematic illustrates how diverse pathological stimuli—including hypoxia, inflammatory cytokines (TNF-α, IL-1β), metabolic stress (ROS), and DAMPs—converge on the IKK complex via upstream pathways (TLR4/MyD88, SphK1/S1P, PERK/eIF2α, and MAPK/ERK) to activate NF-κB. Nuclear translocation of p65/p50 drives transcription of IL-6, TNF-α, MCP-1, HIF-1α, and Cyclin D1, promoting endothelial dysfunction, PASMC proliferation, inflammatory cell recruitment, and extracellular matrix deposition, ultimately leading to vascular remodeling, luminal narrowing, and elevated pulmonary pressure.

### 3.2 BMPR2/NF-κB signaling pathway

BMPR2 mutations represent the most common genetic cause of heritable PAH (HPAH), accounting for approximately 80% of familial cases and 20–25% of idiopathic PAH cases. Importantly, reduced BMPR2 expression is also observed in idiopathic PAH patients without identifiable mutations, suggesting that BMPR2 dysfunction extends beyond monogenic disease (54, 60). Emerging evidence indicates that BMPR2 loss and NF-κB activation are mechanistically intertwined through multiple pathways, forming a bidirectional pathogenic axis that amplifies pulmonary vascular inflammation and remodeling (61). First, BMPR2 deficiency directly promotes NF-κB-dependent inflammatory responses in pulmonary vascular cells. In BMPR-II-deficient PASMCs, the antiproliferative response to TGF-β1 is lost through a Smad-independent mechanism involving aberrant NF-κB activation and enhanced induction of IL-6 and IL-8. Neutralizing antibodies to IL-6 or IL-8 restore the antiproliferative effect of TGF-β1 in HPAH PASMCs (60), indicating that NF-κB-mediated inflammatory cytokine production is a critical mediator of BMPR2 loss-of-function phenotypes (60). Consistent with this, BMPR-II haploinsufficiency in mouse and human PASMCs is associated with increased phospho-STAT3 and enhanced inflammatory cytokine expression after exposure to LPS. Second, BMPR2 deficiency activates the NLRP3/GSDME-mediated pyroptosis pathway in pulmonary arterial endothelial cells, leading to IL-1β release and endothelial injury (62). Knockout of NLRP3 or knockdown of GSDME in animal models alleviates PAH progression, accompanied by improved endothelial integrity, reduced vascular remodeling, and decreased right ventricular systolic pressure. Blocking BMPR2 is sufficient to induce NLRP3 upregulation and release of inflammatory factor IL-1β in pulmonary arterial endothelial cells. Moreover, BMPR2 deficiency can induce GSDME-mediated pyroptosis through NLRP3 activation in animal models, whereas activation of BMPR2 signaling by FK506 or BMP9 reverses these phenotypes. These findings provide direct evidence that loss of BMPR2 signaling promotes endothelial cell pyroptosis by enhancing NLRP3/GSDME signaling, establishing a mechanistic link between BMPR2 dysfunction and NF-κB-associated inflammation (62). Third, NF-κB activation can reciprocally suppress BMPR2 expression, establishing a feedforward loop. The STING-NF-κB axis transcriptionally regulates F2RL3 (63), which in turn activates interferon signaling and represses BMPR2 signaling both *in vitro* and *in vivo (*63). In PH patients and rodent PH models, the cGAS-STING signaling pathway is activated in lung tissues, and genetic knockout or pharmacological inhibition of STING prevents the progression of PH. Mechanistically, STING transcriptionally regulates its binding partner F2RL3 through the STING-NF-κB axis, which activates interferon signaling and represses BMPR2 signaling (63). Additionally, NF-κB-mediated upregulation of miR-130a directly targets BMPR2, creating a positive feedback loop that sustains endothelial dysfunction and vascular remodeling (64).

Clinically and experimentally, the reciprocal relationship between BMPR2 loss and NF-κB activation has been further validated. In Bmpr2^(+/Δ71) rats, tumor-bearing animals exhibited elevated lung IL-1β and NF-κB activation (65). Conditioned media from Bmpr2^(+/Δ71) tumors induced proliferation of pulmonary arterial smooth muscle cells via IL-1β-dependent signaling, while neutralization of IL-1β attenuated this effect (65). These findings identify defective BMPR2 signaling and tumor-associated inflammation as a shared mechanism linking breast cancer and PAH. Importantly, NF-κB activation has been shown to exacerbate, but is not strictly required for, Bmpr2-related pulmonary hypertension. In Bmpr2 mutant mice in which NF-κB activation is genetically blocked, pulmonary hypertension develops indistinguishably from that without the block; however, delivery of a virus causing NF-κB activation strongly exacerbates PH development in Bmpr2 mutant mice. This suggests that NF-κB functions as a critical modifier—or “second hit”—rather than the sole mediator of BMPR2-dependent disease. Collectively, these findings establish BMPR2 loss as both a consequence and a driver of NF-κB activation, positioning NF-κB as a key downstream effector and amplifier of BMPR2-related pathogenesis within a broader pathogenic signaling network. However, NF-κB is a pathogenic amplifier rather than the root genetic driver. Restoring BMPR2 function (e.g., sotatercept, BMP9) is the primary disease-modifying strategy; NF-κB inhibition is conceptually adjunctive—it may dampen the inflammatory “second hit” but cannot substitute for BMPR2 restoration.

### 3.3 TLR4/NF-κB/HIF-1α signaling pathway

Toll-like receptor 4 (TLR4, also known as CD284) is a key innate immune receptor that recognizes pathogen-associated molecular patterns (PAMPs) and damage-associated molecular patterns (DAMPs) (66, 67). It is expressed in both immune cells (monocytes/macrophages, neutrophils, dendritic cells) and vascular cells including HPAECs, PASMCs, and platelets, thereby serving as a critical link between inflammation and pulmonary vascular remodeling.

TLR4 can be activated by multiple DAMPs during PAH progression, including lipopolysaccharide (LPS), high mobility group box 1 (HMGB1), fibronectin, and hyaluronic acid (68, 69). These stimuli activate NF-κB signaling via MyD88- and TRIF-dependent pathways, promoting IκB degradation and inducing transcription of pro-inflammatory cytokines such as IL-6, TNF-α, and MCP-1. Notably, NF-κB also enhances HIF-1α transcriptional activity, establishing a positive feedback loop between inflammatory and hypoxic signaling (42).

The HIF family comprises HIF-1, HIF-2, and HIF-3. In PAH, HIF-1α primarily drives PASMC proliferation, metabolic reprogramming, and apoptosis resistance, whereas HIF-2α is more closely associated with endothelial dysfunction and vascular remodeling (70). Sustained activation of the TLR4/NF-κB/HIF-1α axis under hypoxia and inflammation promotes endothelial dysfunction, EndMT, PASMC proliferation and migration, extracellular matrix deposition, and inflammatory cell recruitment, ultimately leading to intimal thickening, medial hypertrophy, and luminal narrowing, thereby increasing pulmonary vascular resistance. In experimental models, overactivation of this axis correlates with elevated RV systolic pressure (RVSP), mean pulmonary arterial pressure (mPAP), and right ventricular remodeling parameters, including medial wall thickness and RV hypertrophy (71).

Therapeutically, the TLR4/NF-κB/HIF-1α axis has emerged as a promising target for anti-remodeling intervention. For example, cinnamaldehyde, a natural compound, suppresses RVSP and mPAP elevation, attenuates vascular remodeling, reduces inflammatory cytokine release, inhibits hypoxia-induced EndMT, and limits PASMC proliferation (71, 72). Collectively, these findings suggest that targeting the TLR4/NF-κB/HIF-1α signaling network may disrupt the inflammation–hypoxia feedforward loop and reverse pulmonary vascular remodeling, providing a mechanistic basis for future PAH therapies.

Despite substantial evidence supporting the pathogenic role of the TLR4/NF-κB/HIF-1α axis in PAH, several limitations should be considered. First, most available evidence is derived from experimental models, particularly hypoxia-induced and monocrotaline-induced pulmonary hypertension models, which incompletely recapitulate the heterogeneous pathogenesis of human PAH. Whether TLR4 activation represents a universal mechanism across different PAH subtypes remains unclear. Second, TLR4 signaling exhibits complex cell-specific effects. While excessive TLR4 activation in endothelial cells and PASMCs appears to promote inflammation and vascular remodeling, TLR4-mediated innate immune responses may also contribute to tissue repair and host defense, suggesting that complete inhibition of this pathway may have undesirable consequences. Third, although NF-κB and HIF-1α are frequently described as forming a positive feedback loop, the temporal sequence and molecular hierarchy between inflammatory activation and hypoxic adaptation remain insufficiently defined. Future studies using human PAH samples combined with single-cell transcriptomics and spatial multi-omics approaches are needed to determine whether TLR4/NF-κB/HIF-1α activation represents an initiating event or a secondary amplifier during disease progression. Here, NF-κB inhibition is an indirect downstream effect of TLR4 antagonism. Given the failure of systemic TLR4 inhibitors in sepsis, this axis is only therapeutically relevant in hypoxia/inflammation-driven PAH subtypes, and would require pulmonary-targeted delivery to avoid broad immunosuppression.

### 3.4 SphK1/S1P/NF-κB/cyclin D1 signaling pathway

A vital lipid kinase, sphingosine kinase 1 (SphK1) catalyzes the phosphorylation of sphingosine (Sph) to produce the bioactive sphingosine-1-phosphate (S1P), which is implicated in angiogenesis, immune-inflammatory modulation, cell proliferation, and apoptosis resistance (73–75). SphK1 has been shown to be vital for the growth of PASMCs and pulmonary vascular remodeling (75). It is also significantly expressed in a number of human malignancies and pathological vascular disorders. Extracellularly released S1P binds to G protein-coupled receptors (S1P1–5) to initiate downstream signal transduction, including the activation of the NF-κB pathway, which controls cell function and gene expression.

Excessive activation of the SphK1/S1P signaling pathway can encourage the nuclear translocation of the NF-κB p65 subunit under inflammatory or pathogenic stimulation circumstances (76, 77). Nuclear NF-κB drives PASMC cell cycle progression from the G1 phase to the S phase and promotes proliferation by binding to several regulatory sites on the cyclin D1 promoter and directly increasing its transcriptional activity.*In vitro* studies have demonstrated that activation of the SphK1/S1P signaling pathway increases cyclin D1 expression and increases the nuclear activity of NF-κB in PASMCs under hypoxic or inflammatory stimulation; in the MCT-induced PAH rat model, activation of this pathway also correlates with the vascular remodeling phenotype of thickening of the pulmonary artery media and stenosis of the lumen.

As the central hub of SphK1/S1P-mediated vascular remodeling, NF-κB integrates inflammatory signals, stress responses, and metabolic adaptations in addition to controlling the transcription of cell cycle genes. This creates a self-amplifying loop that promotes proliferation, inhibits apoptosis, and increases inflammation. The aberrant activation of the SphK1/S1P/NF-κB/cyclin D1 axis under PAH pathological circumstances might constantly stimulate PASMC proliferation and vascular media hypertrophy, hastening the development of pulmonary vascular remodeling and PAH (78). As a result, this signaling pathway offers prospective targeted treatment approaches for influencing pulmonary vascular remodeling and preventing the aberrant proliferation of PASMCs, in addition to providing a molecular foundation for the investigation of the pathogenesis of PAH.

Although the SphK1/S1P/NF-κB/cyclin D1 pathway provides a mechanistic explanation for PASMC proliferation and vascular remodeling, current evidence remains largely preclinical. Most studies have relied on pharmacological inhibitors or genetic manipulation in cultured PASMCs and rodent models, limiting direct extrapolation to human PAH. In addition, S1P signaling is highly context dependent because different S1P receptors (S1PR1–S1PR5) may exert divergent effects on vascular permeability, immune regulation, and cell survival (79). Therefore, the net consequence of S1P pathway modulation may depend on the specific cellular compartment and disease stage. Moreover, NF-κB activation downstream of S1P is not necessarily restricted to proliferative responses, as it may interact with metabolic pathways, endothelial dysfunction, and immune regulation. Future investigations should clarify the contribution of individual S1P receptor subtypes and determine whether selective rather than global inhibition of S1P/NF-κB signaling provides therapeutic benefits with fewer adverse effects.

### 3.5 PERK/eIF2α/NF-κB signaling pathway

Protein folding, hormone and signaling molecule synthesis, inflammation, metabolism, and endocrine homeostasis are regulated by the endoplasmic reticulum (ER), a key cellular signaling and metabolic hub (80). Endoplasmic reticulum stress (ERS) is induced by accumulation of unfolded or misfolded proteins in the ER lumen, triggered by hypoxia, oxidative stress, Ca²^+^ imbalance, toxins, or viral infections. ERS restores proteostasis by activating the unfolded protein response (UPR), a key adaptive mechanism (81). However, prolonged/chronic ERS contributes to the pathogenesis of PAH and promotes pulmonary vascular remodeling via inflammation and abnormal cell proliferation (82).

Protein kinase R-like endoplasmic reticulum kinase (PERK), inositol-requiring enzyme 1 (IRE1), and activating transcription factor 6 (ATF6) are three ER membrane-resident stress sensors that maintain ER homeostasis through UPR activation (83). Under ER stress, PERK dissociates from the molecular chaperone GRP78, leading to its dimerization and autophosphorylation, thereby activating the PERK signaling cascade. Activated PERK phosphorylates eukaryotic translation initiation factor 2α (eIF2α), resulting in selective translation of stress-responsive genes such as ATF4 while attenuating global protein synthesis (84). In the stationary state, PERK is bound to the molecular chaperone GRP78 and remains in a dormant state. The UPR signaling pathway is activated only when endoplasmic reticulum stress causes GRP78 to separate from PERK (84). Essentially, the PERK/eIF2α axis also links ERS to inflammatory signaling through NF-κB activation. Phosphorylated eIF2α suppresses IκB translation, facilitating IκB degradation via the ubiquitin–proteasome system, which enables NF-κB release and nuclear translocation. Nuclear NF-κB then functions as a transcription factor to induce pro-inflammatory mediators, including TNF-α, IL-6, MCP-1, and ICAM-1, thereby establishing a vascular inflammatory cascade. This inflammatory milieu promotes immune cell recruitment and PASMC migration and proliferation, resulting in medial and intimal thickening and contributing to pulmonary vascular remodeling in PAH. Thus, ERS is tightly linked to inflammation and PASMC proliferation through the PERK/eIF2α/NF-κB axis. NF-κB acts as a key downstream effector in this pathway (85). Therefore, targeting NF-κB-mediated inflammatory and proliferative responses induced by ER stress may represent a promising therapeutic strategy for PAH.

The involvement of the PERK/eIF2α/NF-κB pathway highlights the importance of endoplasmic reticulum stress in PAH; however, several unresolved issues remain. ER stress represents an adaptive cellular response that initially protects cells by restoring protein homeostasis, whereas prolonged activation contributes to pathological remodeling. Therefore, distinguishing beneficial UPR activation from maladaptive chronic ER stress is critical. Thus, future studies should investigate the spatiotemporal regulation of ER stress responses and determine whether selective modulation of pathological PERK/NF-κB activation can preserve physiological stress adaptation.

### 3.6 MAPK/ERK1/2/NF-kB signaling pathway

Extracellular signal-regulated kinases 1 and 2 (ERK1/2), as major components of the mitogen-activated protein kinase (MAPK) family, function as intracellular signaling integrators that respond to growth factors, inflammatory stimuli, oxidative stress, and mechanical stress (86). In pulmonary arterial hypertension (PAH), ERK1/2 is not merely an independent proliferative pathway but serves as an important signaling node that cooperates with NF-κB to regulate inflammatory activation, vascular cell dysfunction, and pulmonary vascular remodeling (87). Accumulating evidence demonstrates that ERK1/2 signaling is persistently activated in pulmonary vascular cells under PAH-associated conditions, including chronic hypoxia, inflammatory stimulation, and oxidative stress. In pulmonary arterial smooth muscle cells (PASMCs), enhanced ERK1/2 activation promotes cell proliferation, migration, and resistance to apoptosis by regulating cell-cycle progression and survival-related transcriptional programs, thereby contributing to medial thickening and increased pulmonary vascular resistance. In pulmonary arterial endothelial cells (PAECs), ERK activation participates in inflammatory responses, endothelial dysfunction, and phenotypic transition, further facilitating vascular remodeling. Importantly, ERK1/2 signaling exhibits extensive crosstalk with NF-κB pathways during PAH progression. Activated ERK1/2 can enhance NF-κB activity through modulation of upstream signaling components, including IKK activation, IκBα degradation, and transcriptional regulation of NF-κB-associated cofactors. Conversely, NF-κB-dependent inflammatory mediators may further amplify ERK signaling, forming a positive feedback loop that sustains vascular inflammation. Through this interaction, ERK/NF-κB signaling promotes transcription of multiple pathogenic genes, including IL-6, TNF-α, MCP-1, cyclin D1, and other inflammatory or proliferative mediators, which collectively contribute to pulmonary vascular remodeling (53).

Several PAH-related studies support the pathological relevance of the ERK/NF-κB axis. Hypoxia-induced ERK activation has been shown to enhance NF-κB-dependent inflammatory responses in PASMCs, promoting proliferation and migration of vascular smooth muscle cells (88). In experimental pulmonary hypertension models, pharmacological inhibition of NF-κB signaling, such as treatment with the IKK inhibitor IMD-0354, attenuates inflammatory cytokine production, PASMC proliferation, pulmonary vascular remodeling, and right ventricular hypertrophy (89). These findings suggest that suppression of ERK/NF-κB-mediated inflammatory amplification may provide therapeutic benefits by simultaneously targeting vascular inflammation and pathological cell activation.

Overall, ERK1/2 functions as an critical component of the NF-κB-centered regulatory network in PAH. Rather than acting as an isolated mitogenic pathway, ERK integrates inflammatory, metabolic, and mechanical signals to reinforce NF-κB-driven vascular remodeling (90). Targeting the ERK/NF-κB axis may therefore represent a potential complementary therapeutic strategy for attenuating pulmonary vascular inflammation and remodeling.

Although ERK1/2 and NF-κB cooperate in promoting pulmonary vascular remodeling, the specific contribution of this axis remains incompletely defined. ERK signaling also maintains endothelial survival and vascular homeostasis, suggesting that systemic ERK inhibition may cause unintended effects (91). Moreover, most studies rely on pharmacological inhibitors with limited specificity, making it difficult to distinguish NF-κB-dependent effects from broader MAPK modulation (91). Future studies using cell-specific genetic approaches and human-derived models are needed to define the therapeutic potential of ERK/NF-κB targeting in PAH. Although there are currently ERK inhibitors available (such as trametinib used for melanoma), their effect on NF-κB is influenced by the extensive regulation of the MAPK pathway and the cytotoxicity of endothelial cells (92). There are currently no validated drug tools, so precise intervention at the ERK/NF-κB interface still belongs to an unproven mechanistic concept rather than a comprehensive inhibition of ERK.

## 4 The regulatory network of NF-κB on RV remodeling in PAH

### 4.1 Cell-type-specific roles of NF-κB in right ventricular remodeling

The transition from adaptive RV hypertrophy to maladaptive remodeling and failure in PAH is a complex, multicellular process initiated by chronic pulmonary vascular pressure overload and involving diverse cell populations within the right ventricle. NF-κB activation is not uniform across these cell types; rather, it exerts fundamentally distinct functions in cardiomyocytes, cardiac fibroblasts, endothelial cells, and infiltrating immune cells. This cell-type specificity determines whether NF-κB signaling promotes protective adaptation or drives pathological remodeling, and has critical implications for therapeutic development.

In cardiomyocytes, NF-κB contributes to hypertrophic remodeling by integrating inflammatory and stress-response signals. NFAT and NF-κB are distinct transcription factor families that cooperate in regulating pathological cardiac hypertrophic responses (93). Chronic pulmonary hypertension-associated pressure overload and neurohumoral stress induce sustained NF-κB activation in the RV, which may contribute to cardiomyocyte hypertrophic remodeling. Pharmacological activation of PPARγ with pioglitazone attenuates PH, RV hypertrophy, cardiomyocyte hypertrophy, and activation of RV hypertrophic signaling, supporting the contribution of inflammatory pathways, including NF-κB-related signaling, to RV remodeling (94). Robinin has been shown to attenuate cardiac hypertrophy in experimental cardiac remodeling models by modulating SIRT1/NF-κB signaling and suppressing oxidative stress and NLRP3 inflammasome activation (94).

The PARP1-PKM2 axis has emerged as a critical integrator linking NF-κB activation to cardiomyocyte dysfunction in RV failure. Increased PARP1-PKM2 signaling has been observed in decompensated RVs from PAH patients and experimental models. Overactivated PARP1 promotes cardiomyocyte dysfunction by favoring PKM2 expression and nuclear function, enhancing glycolytic gene expression, and activating NF-κB-dependent proinflammatory factors. This PARP1-PKM2-NF-κB axis integrates oxidative stress, inflammation, and metabolic dysfunction during pathological RV failure. Pharmacological and genetic inhibition of PARP1, or enforced tetramerization of PKM2, attenuates maladaptive remodeling and improves RV function in experimental models (95). PARP1 inhibitors such as olaparib (NCT03782818) are clinically available in oncology, although their therapeutic potential in PAH-associated RV failure remains investigational. Vitamin D supplementation has also been shown to suppress the Hes1-PARP1 axis while upregulating TNFAIP3, leading to inhibition of NF-κB activation and attenuation of RV hypertrophy.

Unlike pulmonary adventitial fibroblasts, which primarily regulate vascular extracellular matrix remodeling, RV fibroblasts respond to myocardial mechanical stress by regulating interstitial fibrosis and ventricular stiffness. In cardiac fibroblasts, NF-κB drives the transition from quiescent fibroblasts to activated myofibroblasts, promoting extracellular matrix deposition and right ventricular fibrosis. Pulmonary artery banding (PAB)-induced RV pressure-overload models leads to RV dysfunction through NF-κB-mediated inflammation and fibrosis. Periostin (POSTN) expression is increased in RVs of monocrotaline-induced PAH model rats and enhances inducible nitric oxide synthase (iNOS) expression and NO production partially through ERK1/2, JNK, and NF-κB signaling pathways in RV fibroblasts, thereby contributing to RV failure (96). Fibroblast-derived CGRP has been reported to suppress cardiac fibrosis through inhibition of NF-κB activation, suggesting that endogenous negative regulators of NF-κB in fibroblasts may represent protective mechanisms against RV fibrosis.

In cardiac endothelial cells exposed to pressure overload and inflammatory stress, NF-κB activation promotes inflammatory activation, endothelial dysfunction, and impaired angiogenesis, contributing to capillary rarefaction and the transition from compensatory hypertrophy to failure. While direct evidence specifically linking RV endothelial NF-κB to PAH-induced RV failure remains an area of active investigation, NF-κB is known to contribute to RV dysfunction by modulating inflammatory processes in the RV microenvironment.

In infiltrating immune cells, NF-κB activation amplifies RV inflammation through the production of pro-inflammatory cytokines and chemokines. The TWEAK/Fn14 axis represents an NF-κB-associated inflammatory pathway implicated in pressure-overload-induced RV remodeling: Fn14 is significantly upregulated in the right ventricle after PAB, TWEAK-induced collagen synthesis is significantly reduced in Fn14 knockout heart fibroblasts, and Fn14 ablation attenuates RV hypertrophy (97). TLR9 participates in the contributes to PAH development by activating the NF-κB/IL-6 pathway (98). TLR9 inhibition has been reported to attenuate inflammatory responses and improve cardiac remodeling in experimental models. Macrophage accumulation in the right ventricle is a feature of PAH, and NF-κB signaling in macrophages drives the production of IL-1β, IL-6, TNF-α, and MCP-1/CCL2, promoting immune-cell recruitment and establishing a self-sustaining inflammatory microenvironment that accelerates the transition to RV failure (41).

### 4.2 IL-6/STAT3/NF-κB signaling pathway

Interleukin-6 (IL-6) is a 26 kDa secreted protein composed of 184 amino acids, featuring four cysteine residues and two N-glycosylation sites (99, 100). Myeloid cells, including macrophages and dendritic cells, rapidly produce IL-6 upon pathogen recognition via Toll-like receptors (TLRs) (101). Beyond its classical roles in innate and adaptive immunity—including B cell differentiation into plasma cells, antibody production, bone homeostasis, and liver regeneration—IL-6 has emerged as a critical inflammatory mediator in PAH pathogenesis. Elevated circulating IL-6 levels have been consistently observed in PAH patients and correlate with disease severity, hemodynamic impairment, and poor prognosis (102). In experimental models, IL-6 overexpression alone is sufficient to induce pulmonary hypertension, whereas IL-6 knockout attenuates hypoxia-induced PH, establishing a causal role for IL-6 in PAH pathogenesis (103). IL-6 expression is tightly controlled at transcriptional and post-transcriptional levels, with binding sites for NF-κB, NF-IL6, AP1, SP1, and IRF1 in its 5′ cis-regulatory element (104). Importantly, IL-6 functions as a dual upstream activator of both STAT3 and NF-κB pathways: it signals through the IL-6 receptor (IL-6R) and gp130 coreceptor to activate the JAK/STAT3 pathway, while simultaneously engaging NF-κB signaling through IKK complex activation (105).

Signal transducer and activator of transcription 3 (STAT3) is a crucial transcription factor activated downstream of JAK signaling (106). Tissue remodeling, metabolic reprogramming, cell proliferation, and inflammation all depend on it (107). Due to the persistent rise in PVR, individuals with PAH experience prolonged pressure overload in the right ventricle. Inflammatory and neurohumoral factors—including IL-6, TNF-α, platelet-derived growth factor (PDGF), endothelin-1 (ET-1), and angiotensin II (Ang II)—induce sustained phosphorylation of STAT3 at Tyr705 and its nuclear translocation (108). This triggers the transcription of target genes linked to inflammatory responses, anti-apoptosis, and cardiomyocyte hypertrophy. Thus, in PAH, aberrant STAT3 activation is thought to be a key molecular event in the conversion of RV adaptive hypertrophy to decompensatory remodeling (109).

Emerging evidence indicates a close bidirectional regulatory link between STAT3 and NF-κB, forming a crucial inflammatory signaling network that drives right heart remodeling (110). On one hand, TNF-α and oxidative stress promote IκB phosphorylation and degradation through the IKK complex, releasing NF-κB into the nucleus (111). On the other hand, IL-6/JAK signaling continually activates STAT3. Inflammatory and anti-apoptotic genes including IL-6, MCP-1, ICAM-1, COX-2, and Bcl-2 can be expressed more effectively when activated STAT3 and NF-κB work together to engage the transcriptional co-activator CBP/p300 (112). Simultaneously, non-phosphorylated STAT3 can form stable complexes with NF-κB in the nucleus and persistently accumulate there, sustaining chronic inflammatory gene expression and creating a self-amplifying inflammatory feedback loop of STAT3/NF-κB (113). This continuous activation state accelerates RV structural remodeling, causing cardiomyocyte enlargement, fibroblast activation, myocardial interstitial collagen deposition, and ventricular fibrosis (41). In dendritic cells (DCs), IL-6 stimulation alone can activate the IKK complex, promoting IκB phosphorylation and degradation, allowing NF-κB to enter the nucleus and transcribe inflammatory factors such as IL-6, IL-1β, IL-23, and MCP-1, creating a persistent inflammatory microenvironment that directly drives myocardial fibrosis and RV hypertrophy (114, 115). Notably, *in vitro* and animal studies have shown that IL-6/NF-κB stimulation in DCs alone can greatly accelerate RV remodeling, indicating that this axis is essential for PAH right heart remodeling (116). Additionally, proteinase-activated receptor 1 (PAR1) can increase IL-6 and MCP-1 secretion through activation of phosphorylated ERK1/2 and NF-κB, which in turn promotes VSMC proliferation and RV hypertrophy (117). As both an upstream activator of NF-κB and a downstream effector molecule, IL-6 creates a self-amplifying positive feedback loop that ultimately leads to irreversible RV remodeling and heart failure.

Crucially, PKM2 can increase the transcription of NF-κB-dependent inflammatory genes and further stimulate STAT3 activation, establishing a positive feedback loop of PARP1/PKM2/STAT3/NF-κB metabolic-inflammation (95). Continuous stimulation of this system promotes the RV’s progressive transition from compensatory hypertrophy to decompensatory heart failure through increased oxidative stress, cardiomyocyte death, and aberrant activation of cardiac fibroblasts (118). Thus, the STAT3/NF-κB signaling network is a key molecular hub linking metabolic reprogramming, cardiac hypertrophy, and myocardial fibrosis in addition to being an indispensable regulatory axis for the inflammatory response in PAH. The vicious loop of RV remodeling is anticipated to be broken by precise intervention that targets the STAT3/NF-κB signaling axis and its upstream PARP1-PKM2 metabolic network. Olaparib, a PARP1 inhibitor, is now undergoing clinical development (Clinical Trial Number: NCT03782818) and is anticipated to postpone RV remodeling and the advancement of right heart failure by blocking the aberrant activation of STAT3/NF-κB mediated by PARP1 (119). Tocilizumab, a humanized anti-IL-6R monoclonal antibody, has also been evaluated in PAH patients; however, clinical results have been heterogeneous, with only subsets of patients showing benefit, suggesting that IL-6-driven inflammation may be more relevant in specific PAH subtypes (120, 121). Despite these advances, several unresolved questions remain, including whether elevated IL-6 represents a driver or a consequence of RV failure, the relative contribution of classical versus trans-signaling pathways, the identification of reliable biomarkers to predict therapeutic response, and the optimal timing of intervention. These unanswered questions underscore the need for continued mechanistic and translational research to refine therapeutic strategies targeting the IL-6/STAT3/NF-κB axis in PAH.

## 5 The regulatory role of NF-κB in EndMT in PAH

### 5.1 NF-κB orchestrates EndMT in PAH

The highly dynamic process known as EndMT causes ECs to shed their distinctive markers and take on a mesenchymal-like phenotype, gaining the ability to migrate and invade. Although EndMT was first identified during the development of the embryonic heart, it also takes place in adult tissues and plays a role in wound healing, inflammatory reactions, and the pathophysiology of cardiovascular diseases such as atherosclerosis, valvular heart disease, fibroelastic hyperplasia, and PAH (122–124).

Endothelial markers like vascular endothelial cadherin (VE-cadherin), PECAM-1 (CD31), von Willebrand factor (vWF), VEGFR, and Tie-2 are downregulated during EndMT, while mesenchymal proteins like α-smooth muscle actin (α-SMA), fibronectin, and vimentin are upregulated (125–127). ECs also separate from nearby cells and rearrange their actin cytoskeleton to form protrusions. Transcription factors including Snail, Slug, Zeb1/2, and Twist-1 coordinate the activation of mesenchymal programs and the suppression of endothelial genes, causing ECs to change from a compact cobblestone-like structure to a spindle-like morphology (128, 129).

Recent research links endothelial dysfunction to intimal thickening, extracellular matrix deposition, and vascular stiffness, implicating EndMT as a major factor in pulmonary vascular remodeling in PAH. Crucially, NF-κB has been found to be a key regulator of EndMT, combining mechanical and inflammatory inputs to encourage EC transcriptional reprogramming (130). Therefore, a viable treatment approach for reducing vascular remodeling in PAH is to target the NF-κB pathway to modulate EndMT. This idea connects previous considerations of NF-κB-mediated vascular and RV remodeling to possible therapeutic strategies by proposing a direct molecular link between NF-κB-driven inflammation in pulmonary vessels and structural vascular alterations.

**Figure 2:**
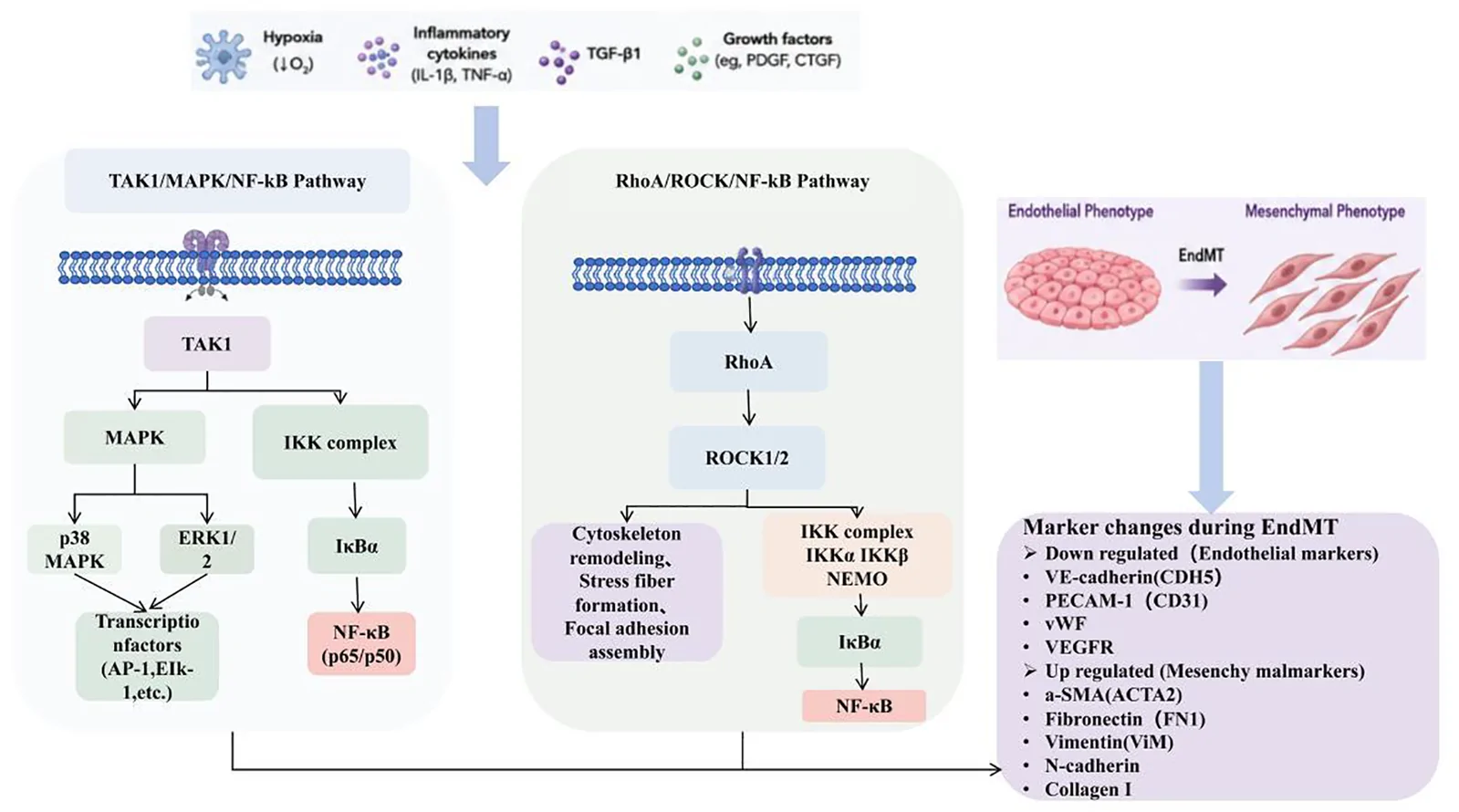
NF-κB integrates inflammatory and mechanical signals to drive endothelial–mesenchymal transition (EndMT) in PAH.

Despite substantial *in vitro* and transcriptomic evidence linking NF-κB to EndMT, the *in vivo* contribution of this process to PAH remains debated. Genetic lineage-tracing studies have yielded inconsistent results, with some showing limited endothelial-derived mesenchymal cells in vascular lesions, suggesting that resident fibroblasts, rather than EndMT, may be the primary source of myofibroblasts (131). Moreover, *in vitro* and transcriptomic approaches cannot fully recapitulate the complex multicellular interactions and mechanical microenvironment *in vivo*; the ‘EndMT’ observed in such settings may reflect partial cellular plasticity rather than complete phenotypic conversion (131). Thus, distinguishing direct cell transdifferentiation from paracrine-mediated indirect remodeling is critical when interpreting EndMT data (132). These caveats do not diminish the relevance of NF-κB in endothelial dysfunction and vascular remodeling, but they underscore the need for refined *in vivo* lineage-tracing and functional studies to define the true extent and therapeutic relevance of EndMT in PAH. The molecular events of EndMT involve multiple signaling cascades, with NF-κB acting as a key transcription factor that integrates inflammatory and mechanical cues. **Figure 2** illustrates how NF-κB drives endothelial phenotypic switching through the TAK1/MAPK and RhoA/ROCK axes.

This schematic illustrates how hypoxia, inflammatory cytokines (IL-1β/TNF-α), TGF-β1, and growth factors (PDGF/CTGF) converge on the TAK1/MAPK/NF-κB and RhoA/ROCK/NF-κB axes to activate NF-κB. Upon nuclear translocation, NF-κB orchestrates EndMT by downregulating endothelial markers (VE-cadherin, CD31) and upregulating mesenchymal markers (α-SMA, vimentin), leading to extracellular matrix deposition, vascular stiffening, and intimal thickening. This figure emphasizes the mechanistic role of NF-κB as a central hub linking extracellular stimuli to endothelial phenotypic switching, focusing on the cellular events underlying vascular wall pathology.

Transforming growth factor-β-activated kinase 1 (TAK1) is a crucial serine/threonine kinase that plays vital roles in immunological homeostasis, tissue remodeling, and organ development. It integrates signaling pathways associated to inflammation, immunity, and stress (133, 134). There is growing evidence that TAK1 expression and phosphorylation levels are significantly increased in models of PAH caused by monocrotaline (MCT), indicating that aberrant TAK1 activation plays a role in pulmonary vascular remodeling (135). The TAK1/MAPK/NF-κB axis is one of the downstream signaling cascades that has been identified as a critical regulatory route influencing the EndMT (136).

TGF-β1, inflammatory cytokines (such as IL-1β and TNF-α), and other growth factors stimulate TAK1, which then rapidly activates and phosphorylates downstream MAPK signaling molecules, such as p38 MAPK and ERK1/2, while also activating the canonical NF-κB pathway through IKK-mediated phosphorylation and degradation of IκBα. In order to coordinate endothelial phenotypic reprogramming, the released NF-κB p65/p50 complex translocates into the nucleus and works with MAPK-dependent transcription factors to induce the expression of pro-survival genes, inflammatory mediators, and transcriptional regulators related to EndMT (136).

In HPAECs, persistent activation of the TAK1/MAPK/NF-κB signaling cascade induces significant phenotypic changes. Along with the downregulation of endothelial markers like VE-cadherin and the upregulation of mesenchymal markers like α-SMA, these cells gradually lose their distinctive cobblestone morphology and take on an elongated spindle-shaped appearance, signifying the start and progression of EndMT. The resultant mesenchymal-like cells have increased extracellular matrix formation, profibrotic activity, and migratory ability, which eventually aid in pulmonary vasculature remodeling and intimal thickening (137–139).

Crucially, NF-κB serves as this signaling network’s primary inflammatory amplifier. A prolonged inflammatory milieu is created in the pulmonary vasculature by activated NF-κB, which significantly increases the transcription of several pro-inflammatory cytokines and chemokines, such as IL-1β, IL-6, TNF-α, and MCP-1. Through autocrine and paracrine pathways, these inflammatory mediators further boost TAK1 activation, creating a TAK1–NF-κB positive feedback loop that continuously worsens endothelial damage, speeds up the course of EndMT, and encourages permanent pulmonary vascular remodeling. In order to avoid EndMT and reduce pulmonary vascular remodeling in PAH, pharmacological suppression of the TAK1/MAPK/NF-κB signaling cascade, specifically targeting NF-κB-mediated inflammatory amplification, may be a promising treatment approach (136).

Although TAK1 has been implicated as an upstream regulator of NF-κB-mediated EndMT, its specific contribution remains unclear. TAK1 integrates multiple pathways, including TGF-β, MAPK, and inflammatory signaling, making it difficult to distinguish NF-κB-dependent effects from other downstream mechanisms. Moreover, the extent of EndMT involvement in human PAH remains controversial, as endothelial plasticity may represent a spectrum rather than complete phenotypic conversion. Current evidence is largely derived from experimental models, and validation in human PAH tissues using lineage-tracing and single-cell approaches is needed.

### 5.2 RhoA/ROCK/NF-κB signaling pathway

Actin cytoskeleton dynamics, cell polarity, migration, and contractility are all regulated by Ras homolog family member A (RhoA), a member of the Rho family of small GTPases (140, 141). Stress fiber formation, focal adhesion assembly, and cytoskeletal remodeling are all regulated by GTP-bound RhoA’s interactions with its primary downstream effectors, Rho-associated coiled-coil-containing protein kinases (ROCK1 and ROCK2) (142, 143). There is growing evidence that pulmonary vascular remodeling and endothelial dysfunction in PAH are caused by inappropriate activation of the RhoA/ROCK signaling system, namely by encouraging EndMT (144).

Activation of the RhoA/ROCK pathway causes significant cytoskeletal reorganization and mechanical signaling in PAECs under pathological conditions like hypoxia, inflammation, and TGF-β1 stimulation. This leads to the loss of endothelial integrity and the acquisition of mesenchymal traits. Endothelial markers like VE-cadherin and CD31 are gradually downregulated during this process, while mesenchymal markers like fibronectin, vimentin, and α-SMA are significantly upregulated along with increased migratory and extracellular matrix-producing abilities. These changes in phenotype hasten pulmonary vascular remodeling and intimal thickening (125, 145).

NF-κB is an critical downstream inflammatory effector of the RhoA/ROCK pathway. The IKK complex is phosphorylated by ROCK activation, which causes IκBα breakdown and nuclear translocation of NF-κB p65/p50 heterodimers (146). This starts the transcription of many pro-inflammatory mediators, such as IL-1β, IL-6, TNF-α, MCP-1, and ICAM-1. Endothelial phenotypic reprogramming is ultimately driven by persistent NF-κB activation, which also intensifies inflammatory signaling and collaborates with TGF-β-dependent transcriptional programs to increase EndMT-associated transcription factors, such as Snail, Slug, Twist1, and Zeb1/2. In the meantime, RhoA activation is further stimulated by inflammatory cytokines released downstream of NF-κB through autocrine and paracrine processes, creating a RhoA/ROCK/NF-κB positive feedback circuit that sustains endothelial injury and EndMT progression.

According to recent research, magnesium lithospermate B significantly reduces hypoxia-induced PAH by inhibiting the RhoA/ROCK/NF-κB signaling cascade, which suppresses EndMT (147). The RhoA/ROCK/NF-κB signaling network is a promising therapeutic target for preventing EndMT and reversing pulmonary vascular remodeling in PAH. Pharmacological inhibition of this pathway restores endothelial marker expression, decreases mesenchymal transition, and alleviates pulmonary vascular remodeling.

Although the RhoA/ROCK/NF-κB pathway links mechanical stress, inflammation, and EndMT in PAH, its therapeutic potential remains uncertain. RhoA/ROCK signaling is also essential for vascular tone regulation and endothelial homeostasis, raising concerns regarding systemic inhibition. Moreover, the specific contribution of NF-κB-dependent mechanisms relative to NF-κB-independent ROCK effects remains unclear. Limited clinical evidence further restricts the evaluation of whether ROCK targeting can achieve disease modification beyond hemodynamic improvement.

## 6 NF-κB-mediated epigenetic regulation in PAH

Growing genomic and transcriptomic evidence suggests that epigenetic changes have a significant impact on PAH in addition to genetic abnormalities. Due to their capacity to regulate gene transcription and post-transcriptional expression, non-coding RNAs—in particular, microRNAs (miRNAs) and long non-coding RNAs (lncRNAs)—have become essential regulators of pulmonary vascular remodeling. Beyond the well-characterized miRNA and lncRNA networks, recent advances have highlighted the critical role of chromatin-level and RNA-level modifications in controlling NF-κB activation and its downstream inflammatory programs in PAH. These epigenetic regulatory layers operate through three interconnected mechanisms: (1) DNA methylation and hydroxymethylation, which modulate the accessibility of NF-κB binding sites and the expression of NF-κB regulatory genes (148); (2) histone acetylation, which governs chromatin architecture at inflammatory enhancers and promoters (149); (3) histone methylation, which establishes permissive or repressive chromatin states at NF-κB target loci (47). Collectively, these chromatin-modifying mechanisms, together with non-coding RNAs, form an integrated epigenetic regulatory network that controls NF-κB-driven inflammation and vascular remodeling in PAH. There is growing evidence that NF-κB serves as a master upstream regulator of these epigenetic networks. As a result, focusing on NF-κB-mediated epigenetic regulation has emerged as a promising treatment approach for PAH (150).

### 6.1 NF-κB/miRNA regulatory network

Through complementary binding to the 3′untranslated region (3′-UTR) of target messenger RNAs, miRNAs, endogenous single-stranded non-coding RNAs made up of roughly 20–22 nucleotides, control gene expression by either translational repression or mRNA destruction (151, 152). Numerous biological processes, including cell proliferation, differentiation, apoptosis, inflammation, and metabolic control, have been linked to aberrant miRNA expression. These activities all contribute to pulmonary vascular remodeling in PAH (153).

One of the key mediators of hypoxia-induced PAH among the NF-κB-regulated miRNAs is miR-335-3p. NF-κB directly stimulates the transcription of miR-335-3p under persistent hypoxic circumstances, leading to its significant increase. By preventing the proliferation of PASMC, apelin receptor (APJ), a verified downstream target of miR-335-3p, contributes to the preservation of pulmonary vascular homeostasis (154, 155). By directly interacting with APJ’s 3′-UTR, elevated miR-335-3p inhibits APJ expression, which encourages PASMC proliferation and pulmonary vascular remodeling. These results imply that the NF-κB/miR-335-3p/APJ signaling axis is a viable therapeutic target and a key molecular mechanism driving hypoxia-induced PAH (154).

The abnormal expression of miR-130a, another characteristic NF-κB-dependent miRNA, has been linked to PAH and a number of inflammatory conditions (156). While miR-130a directly suppresses the expression of bone morphogenetic protein receptor type 2 (BMPR2), a critical anti-remodeling receptor in pulmonary endothelial cells, TGF-β1-induced NF-κB activation dramatically increases miR-130a expression in pulmonary microvascular endothelial cells. Endothelial dysfunction, EndMT, and vascular remodeling are all facilitated by loss of BMPR2 signaling (117, 157). Additionally, elevated miR-130a increases the transcriptional activity of NF-κB, creating a positive feedback loop between NF-κB and miR-130a that intensifies inflammatory signaling and endothelial damage. In order to avoid pulmonary vascular remodeling and restore endothelial homeostasis, pharmacological disruption of the NF-κB/miR-130a/BMPR2 axis may offer a potential approach (64).

### 6.2 NF-κB/lncRNA regulatory network

Transcripts longer than 200 nucleotides that control gene expression by chromatin remodeling, transcriptional regulation, RNA sponging, and protein interaction are known as lncRNAs (158–160). According to recent research, lncRNAs play a significant role in pulmonary vasculature remodeling by influencing inflammatory reactions and NF-κB signaling (161).

Among these compounds, lncRNA CPS1-IT1 has been identified as a protective regulator in PAH related with hypoxia (162). CPS1-IT1 attenuates inflammatory activation by suppressing transcriptional activity mediated by HIF-1α and lowering the generation of IL-1β. Crucially, CPS1-IT1 suppresses the production of several pro-inflammatory cytokines and prevents NF-κB nuclear translocation by blocking the phosphorylation of IκBα. CPS1-IT1 reduces pulmonary vascular inflammation and remodeling by concurrently inhibiting the HIF-1α/IL-1β/NF-κB signaling cascade, indicating that restoring CPS1-IT1 expression may be a potential epigenetic approach for the treatment of PAH linked to obstructive sleep apnea (163, 164).

A growing body of research suggests that NF-κB plays a key role in the PAH epigenetic regulatory network. NF-κB creates several positive feedback circuits that sustain inflammation, EndMT, pulmonary vascular remodeling, and disease progression by regulating the transcription of disease-associated miRNAs and lncRNAs while also being reciprocally regulated by these non-coding RNAs. Thus, therapeutic methods that target NF-κB-centered epigenetic signaling networks—such as the NF-κB/miR-335-3p/APJ, NF-κB/miR-130a/BMPR2, and HIF-1α/IL-1β/NF-κB/CPS1-IT1 axes—may offer novel ways to treat PAH precisely in the future.

### 6.3 DNA methylation and hydroxymethylation in NF-κB regulation

DNA methylation at CpG islands is catalyzed by DNA methyltransferases (DNMTs), including DNMT1 (maintenance methylation) and DNMT3A/3B (*de novo* methylation), and is reversed by ten-eleven translocation (TET) enzymes (TET1, TET2, TET3) through active demethylation. Aberrant DNA methylation patterns have been consistently observed in PAH and are closely linked to vascular remodeling and pulmonary vascular dysfunction. Multiple lines of evidence indicate that DNA methylation directly regulates NF-κB signaling in PAH through at least two distinct mechanisms. First, promoter hypermethylation of NFKBIA (the gene encoding IκBα) can silence this critical negative regulator of NF-κB, thereby lowering the threshold for NF-κB activation and promoting sustained inflammatory signaling. Although direct evidence in PAH remains limited, this mechanism has been well established in other inflammatory diseases and represents a plausible pathway requiring further investigation (165). Second, DNMT-mediated hypermethylation of genes encoding antioxidant and anti-inflammatory proteins, such as superoxide dismutase 2 (SOD2), contributes to oxidative stress and enhances NF-κB-dependent inflammatory responses. DNMT1 and DNMT3B have been specifically implicated in this process, linking DNA methylation to NF-κB-driven endothelial dysfunction and PASMC proliferation (166, 167).

TET2 has emerged as a particularly important epigenetic regulator in PAH. Loss-of-function mutations in TET2 have been identified in patients with idiopathic PAH and connective tissue disease-associated PAH. Conditional downregulation of TET2 in hematopoietic and endothelial cells leads to spontaneous PAH development, characterized by increased inflammation, PASMC proliferation, endothelial dysfunction, and adverse pulmonary vascular remodeling (168). Mechanistically, TET2 deficiency induces a state of chronic inflammation that activates NF-κB signaling and promotes the expression of pro-inflammatory cytokines such as IL-1β, thereby contributing to disease progression (148). These findings position TET2 as a protective epigenetic regulator whose loss exacerbates NF-κB-mediated inflammation in PAH (169).

### 6.4 Histone acetylation and NF-κB transcriptional control

Histone acetylation, mediated by histone acetyltransferases (HATs) and removed by histone deacetylases (HDACs) and sirtuins (SIRTs), plays a pivotal role in regulating chromatin accessibility at NF-κB target genes. The balance between acetylation and deacetylation determines the transcriptional output of NF-κB-dependent inflammatory programs in pulmonary vascular cells.

Upon nuclear translocation, NF-κB (predominantly the p65/p50 heterodimer) recruits HATs such as p300 and CBP to the promoters of pro-inflammatory and pro-remodeling genes, including IL6, TNF, MCP-1, MMP9, and VEGF. p300/CBP-mediated histone acetylation (particularly H3K27ac) relaxes chromatin structure and facilitates transcriptional elongation. In PAH, increased p300/CBP activity has been linked to cardiomyocyte hypertrophy and the pro-proliferative, apoptosis-resistant phenotype of PASMCs, suggesting that p300/CBP-dependent NF-κB acetylation contributes to both pulmonary vascular and right ventricular remodeling (170).

Conversely, HDACs and sirtuins restrain NF-κB transcriptional activity through deacetylation of both histones and the RelA/p65 subunit itself. SIRT1, an NAD^+^-dependent deacetylase, directly deacetylates RelA/p65 at lysine 310, thereby inhibiting NF-κB-dependent transcription and sensitizing cells to TNFα-induced apoptosis (171). In PAH, reduced SIRT1 expression or activity would be expected to enhance RelA/p65 acetylation and amplify NF-κB-driven inflammatory responses (172). SIRT6, another member of the sirtuin family, has been shown to regulate the NF-κB pathway through post-translational modification of the histone methyltransferase SUV39H1, revealing an intricate crosstalk between acetylation and methylation pathways (173).

Bromodomain and extra-terminal (BET) proteins, particularly BRD4, function as epigenetic “readers” that recognize acetylated histones and facilitate the recruitment of transcriptional machinery to NF-κB target genes (174). In PAH patients, nuclear staining of BRD2 and BRD4 is significantly increased in lung vascular endothelial and smooth muscle cells compared with controls (175). BET proteins are required for the expression of a subset of NF-κB-induced inflammatory genes, and the BET inhibitor JQ1 suppresses TNFα-driven IL-6 and CXCL8/IL-8 release in human pulmonary microvascular endothelial cells and pulmonary artery smooth muscle cells. Notably, JQ1 exhibits distinct cell-specific efficacy—greater on IL-6 release in endothelial cells and on CXCL8/IL-8 release in smooth muscle cells—suggesting that BET-dependent NF-κB regulation is cell-type-specific (149). These findings support BET proteins as promising therapeutic targets for PAH.

### 6.5 Histone methylation in NF-κB regulation

Histone methylation, mediated by histone methyltransferases and reversed by histone demethylases, establishes chromatin states that either permit or repress NF-κB-dependent transcription. Several histone methylation marks and their corresponding enzymes have been implicated in PAH pathogenesis (176).

Enhancer of zeste homolog 2 (EZH2), the catalytic subunit of Polycomb repressive complex 2, mediates trimethylation of histone H3 at lysine 27 (H3K27me3), a repressive chromatin mark. EZH2 expression and H3K27me3 levels are elevated in PAH-PASMCs (177, 178). EZH2-catalyzed H3K27me3 has been shown to upregulate glutamine metabolism enzymes GLS and GLUD1, linking epigenetic regulation to metabolic reprogramming in PAH (176). Importantly, IL-6-driven STAT3 activation recruits EZH2 to the promoters of anti-angiogenic genes such as THBS1, leading to H3K27me3-mediated transcriptional silencing (176). Given the established crosstalk between STAT3 and NF-κB in PAH, EZH2 may represent an additional layer through which inflammatory signals reinforce NF-κB-driven vascular remodeling.

SUV39H1, a histone methyltransferase that catalyzes H3K9me3 (another repressive mark), has been linked to NF-κB regulation through its interaction with SIRT6. SIRT6 binds to SUV39H1 and induces monoubiquitination in its PRE-SET domain, thereby regulating NF-κB pathway activity. This SIRT6-SUV39H1 axis exemplifies the intricate interplay between different classes of epigenetic modifiers in controlling NF-κB signaling (173).

SETD7 (also known as SET7/9), a lysine methyltransferase, directly methylates the NF-κB subunit RelA/p65 at lysine 37, promoting pro-inflammatory signaling (179). SETD7-mediated methylation of RelA/p65 enhances NF -κB-dependent inflammatory responses, leading to increased inflammation, oxidative stress, and tissue damage (180). Pharmacological inhibition of SETD7 attenuates TNFα-induced inflammation in vascular endothelial cells, identifying SETD7 as a potential therapeutic target for inflammatory-driven vascular dysfunction. Although direct evidence in PAH is limited, the established role of SETD7 in NF-κB activation and vascular inflammation suggests that this pathway warrants further investigation in the PAH context.

The emerging relationship between NF-κB and non-coding RNA networks provides new insights into epigenetic regulation in PAH; however, current evidence should be interpreted cautiously. Most identified miRNAs and lncRNAs have been characterized in specific experimental conditions, and their expression patterns may differ among species, disease stages, and cellular compartments. Moreover, many studies demonstrate association rather than direct causality between altered non-coding RNA expression and NF-κB activation. The biological functions of individual miRNAs or lncRNAs may also vary depending on their interacting targets and cellular context. Although RNA-based therapies represent an attractive strategy, challenges including delivery efficiency, off-target effects, and immune activation remain major barriers. Future studies integrating single-cell transcriptomics, spatial transcriptomics, and functional validation in human PAH tissues are required to identify clinically relevant NF-κB-associated epigenetic signatures. Beyond histone modifications, RNA-level epitranscriptomic changes—such as m6A methylation—also modulate NF-κB activity. **Table 3** summarizes the major epitranscriptomic regulators and their effects on NF-κB signaling in PAH.

**Table 3:** Epitranscriptomic regulation of NF-κB signaling in PAH.

Regulatory category	Regulatory component	Mechanistic link with NF-κB signaling	Major affected cell type(s)	Representative NF-κB downstream targets	Functional consequences	Evidence in pulmonary vascular remodeling	Evidence in right ventricular remodeling
Chromatin and epigenetic regulators	BRD4 (BET family protein)	Functions as a chromatin reader that facilitates NF-κB-dependent enhancer activation and inflammatory gene transcription	PASMCs, inflammatory cells	IL-6, inflammatory cytokines, proliferation-associated genes	PASMC proliferation and inflammatory phenotype switching	Moderate	Limited
Chromatin and epigenetic regulators	SIRT1	Deacetylates NF-κB p65, regulating transcriptional activity and inflammatory responses	Cardiomyocytes, endothelial cells, immune cells	TNF-α, IL-6, inflammatory genes	Regulation of inflammation, oxidative stress, and cardiac remodeling	Emerging	Moderate
Chromatin and epigenetic regulators	SIRT6	Modulates chromatin accessibility and suppresses NF-κB-dependent inflammatory transcription	Endothelial cells, cardiomyocytes	NF-κB inflammatory targets	Protection against endothelial dysfunction and fibrosis	Emerging	Emerging
Non-coding RNA regulators	miR-130a	NF-κB activation induces miR-130a expression, which suppresses BMPR2 signaling and reinforces inflammatory remodeling	PAECs, PASMCs	BMPR2 suppression; secondary NF-κB amplification	Endothelial dysfunction, impaired BMP signaling, vascular remodeling	Strong	Limited
Non-coding RNA regulators	miR-21	NF-κB-associated inflammatory signaling increases miR-21 expression, regulating vascular remodeling-related pathways	PASMCs, fibroblasts	STAT3, inflammatory cytokines, genes, inflammatory mediators	PASMC proliferation, fibrosis, vascular remodeling	Moderate	Emerging
Non-coding RNA regulators	lncRNA CPS1-IT	Modulates HIF-1α/IL-1β/NF-κB inflammatory axis	Vascular cells	IL-1β, inflammatory mediators	Cell proliferation, EndMT, extracellular matrix remodeling	Emerging	Limited
Epitranscriptomic regulators	FTO (m6A demethylase)	Alters mRNA m6A modification and regulates TNF-α/NF-κB inflammatory signaling programs	PAECs, immune cells	TNF-α/NF-κB-associated inflammatory genes	Endothelial inflammation, immune-cell recruitment, pulmonary vascular remodeling	Emerging–Moderate	Unknown
Epitranscriptomic regulators	METTL3/METTL14/WTAP (m6A writers)	m6A deposition regulates stability and translation of inflammatory transcripts and may modulate NF-κB pathway components	Immune cells, vascular cells	NF-κB-associated inflammatory transcripts	Regulation of inflammatory activation and stress adaptation	Emerging	Unknown
Epitranscriptomic regulators	YTHDF/YTHDC family (m6A readers)	Recognizes m6A-modified transcripts and controls RNA stability, translation, and inflammatory responses	Immune cells, vascular cells	Cytokine-related transcripts, NF-κB-associated genes	Post-transcriptional regulation of inflammatory phenotypes	Emerging	Unknown

## 7 Therapeutic strategies targeting NF-κB signaling in PAH

NF-κB has become one of the most appealing therapeutic targets for PAH due to its indispensable role in coordinating pulmonary vascular inflammation, endothelial dysfunction, EndMT, smooth muscle cell proliferation, extracellular matrix remodeling, and RV maladaptive remodeling. Inhibition of NF-κB signaling has the potential to concurrently attenuate various degenerative processes implicated in disease progression, hence offering a disease-modifying therapy strategy, in contrast to existing vasodilator medications that primarily lower PVR.

Natural bioactive substances, synthetic small-molecule inhibitors, epigenetic regulators, biological therapies, and non-pharmacological interventions are the main categories of current NF-κB-targeted interventions. By suppressing NF-κB-dependent inflammatory signaling, a variety of natural products, such as cinnamaldehyde, thymoquinone, formononetin, grape seed procyanidins, allicin, betaine, sophocarpidine, and alginate oligosaccharides, have shown notable protective effects in experimental PAH models (53, 181–185). These substances alleviate pulmonary vascular remodeling, improve RV hypertrophy and fibrosis, inhibit endothelial dysfunction and EndMT, and decrease the proliferation of PASMC. This suggests that naturally derived NF-κB inhibitors could be a significant source of future anti-remodeling treatments.

A number of synthetic small-molecule inhibitors that target NF-κB upstream regulators have demonstrated promising efficacy in addition to natural drugs. Pharmacological suppression of ERK1/2/NF-κB, p38MAPK/NF-κB, RhoA/ROCK/NF-κB, HSP90/NF-κB, and NF-κB/IL-6 signaling substantially decreases PASMC proliferation, inflammatory cytokine production, macrophage activation, and pulmonary vascular remodeling. IMD-0354, nicorandil, KR36676, BET inhibitors, and HSP90 inhibitors are examples of representative agents that have shown the capacity to concurrently improve RV remodeling and pulmonary hemodynamics, suggesting that upstream blockade of NF-κB signaling may offer more extensive therapeutic benefits than focusing on specific downstream mediators (89, 149, 186, 187).

However, what we wish to emphasize here is that although IKKβ inhibitors (such as IMD-0354) show good efficacy *in vitro* experiments, direct inhibition of NF-κB still faces significant obstacles in transformation (89). Firstly, NF-κB is crucial for host defense and liver cell survival; systemic inhibition would increase infection susceptibility and may exacerbate liver damage (188). Secondly, there are significant differences in the functions of IKKα and IKKβ - IKKβ mainly participates in innate immunity, while IKKα regulates adaptive immunity and lymphoid organ development, making selective inhibition rather difficult (189). Thirdly, due to limited efficacy and toxicity dose restrictions, broad-spectrum NF-κB inhibitors have not achieved success in other chronic inflammatory diseases (190). Fourthly, although the NF-κB decoy oligonucleotides delivered by nanoparticles show potential *in vitro* in the PAH model, issues such as delivery efficiency, target cell specificity, and long-term stability have not been resolved (191). Therefore, future research should prioritize cell-specific targeting strategies, such as nanoparticle-based pulmonary administration or antibody-drug conjugates, rather than relying on systemic NF-κB blockade.

NF-κB-directed therapy has been further extended into the field of precision medicine by recent developments. Long non-coding RNAs and miRNAs are examples of epigenetic regulators that control NF-κB transcriptional activity and create reciprocal regulatory loops that affect vascular remodeling and endothelial plasticity. While modulation of the NF-κB/miR-335-3p/APJ and NF-κB/miR-130a/BMPR2 axes effectively attenuates pulmonary vascular remodeling and EndMT, restoration of protective lncRNA CPS1-IT1 expression suppresses the HIF-1α/IL-1β/NF-κB cascade, underscoring the therapeutic potential of RNA-based interventions.

Additionally, it has been demonstrated that new biological therapies, such as immune modulation and induced pluripotent stem cell (iPSC)-based treatment, as well as gaseous signaling molecules like hydrogen sulfide (H2S), can reduce pulmonary vascular inflammation and prevent NF-κB activation (192). It’s interesting to note that non-pharmacological interventions like fitness training can improve RV remodeling and suppress TWEAK/NF-κB signaling, indicating that lifestyle-related interventions may potentially be used to decrease NF-κB (193).

**Figure 3:**
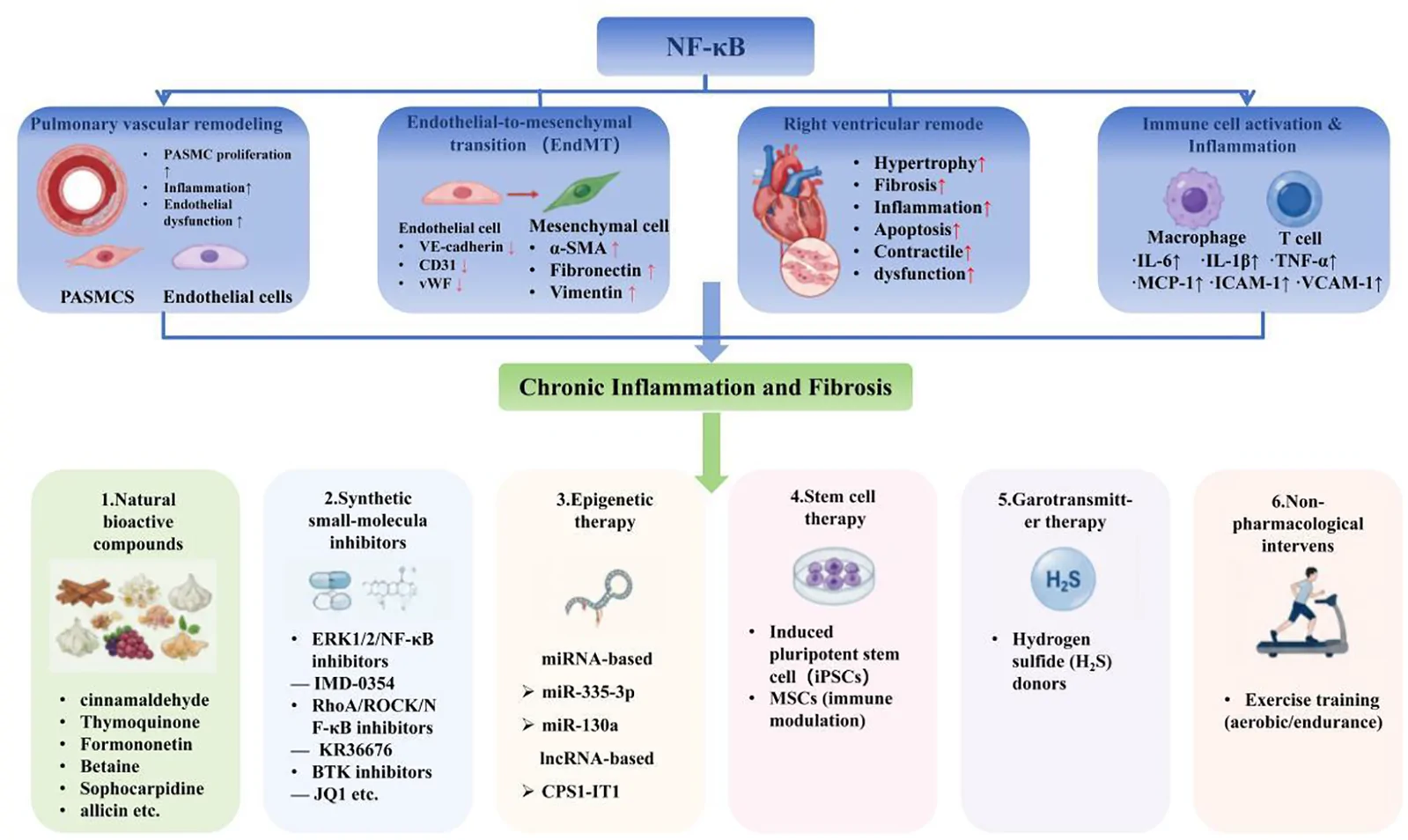
NF-κB as a therapeutic hub in PAH and multi-level intervention strategies.

A growing body of research indicates that NF-κB integrates inflammatory signaling, metabolic reprogramming, EndMT, pulmonary vascular remodeling, and RV remodeling in PAH as a convergent therapeutic node, coordinating multiple pathogenic networks across diverse vascular and cardiac cell types. To maximize efficacy while minimizing systemic immunosuppression, future therapeutic development should prioritize strategies that enable cell-specific modulation of NF-κB signaling. Several emerging approaches hold promise in this regard. Nanoparticle-based drug delivery systems, leveraging controlled release and pulmonary endothelium targeting, can achieve precise localization to the pulmonary vasculature while minimizing systemic side effects (194–196). For instance, rod-shaped nanococrystals designed for pulmonary-endothelium targeting have been shown to ameliorate experimental PH by suppressing the ET-1/NF-κB/ICAM-1/TNF-α/IL-6 pathway. RNA-based therapeutics, including NF-κB decoy oligonucleotides delivered via nanoparticles, have demonstrated efficacy in preventing monocrotaline-induced PAH by blocking NF-κB activation in the lung (191). Cell-specific targeting strategies informed by single-cell transcriptomic and spatial transcriptomic analyses of human PAH lungs—which have revealed distinct cell populations and molecular markers driving vascular remodeling—may enable precise intervention in pathogenic cell subsets such as activated endothelial cells, proliferative PASMCs, and pro-inflammatory macrophages (4, 197). Ultimately, these approaches—whether used individually or in combination—may yield a new class of disease-modifying treatments capable of reversing pulmonary vascular remodeling and improving long-term outcomes for PAH patients. Given the pivotal role of NF-κB in multiple pathogenic processes, targeting this pathway has become a major focus of disease-modifying therapy. **Figure 3** outlines six categories of intervention strategies centered on NF-κB, illustrating their proposed actions on inflammation, remodeling, and cardiac protection. Among the strategies outlined above, several agents have entered clinical trials.**Table 4** summarizes the current clinical status, primary results, and limitations of NF-κB-related inhibitors in PAH patients.

**Table 4:** Current clinical research on NF-κB-related inhibitors in PAH.

Medication	Mechanism of action	Clinical stage	PH type	Main results	Remark
Bardoxolone methyl (CDDO-Me)	Activate Nrf2 and indirectly inhibit the NF-κB inflammatory pathway	Phase II(LARIAT), Phase III(CATALYST, Termination)	IPAH, CTD-PAH, etc.	Some patients showed improvement in 6MWD, but the subsequent development was terminated.	One of the currently most mature indirect NF-κB regulatory drugs
Olaparib	Inhibit PARP1 and reduce the inflammatory signals of STAT3/NF-κB	Phase I/II exploration	PAH	The main evaluation focuses on the changes in PVR. Currently, it is in the stage of mechanism verification.	Not a direct NF-κB inhibitor, but it blocks the PARP1-PKM2-STAT3-NF-κB axis
Dimethyl fumarate (DMF)	Activate Nrf2 and directly inhibit NF-κB (p65)	There is no specific RCT (randomized controlled trial)	Multiple sclerosis is now available on the market.	PAH has only sufficient evidence from animal studies	High clinical translational potential
Tocilizumab	Block IL-6R, and downstream inhibit the amplification of NF-κB inflammation	Phase II exploration	IPAH	The therapeutic effect is limited, and only some inflammatory subtypes may benefit from it.	Belongs to the downstream immune regulation of NF-κB

This overview places NF-κB at the center of PAH pathogenesis, integrating upstream signals to drive pulmonary vascular remodeling, EndMT, right ventricular maladaptation, and immune-inflammatory cascades. Around this NF-κB network, six categories of therapeutic approaches are summarized: natural bioactive compounds, small-molecule inhibitors, epigenetic modulators (miRNAs/lncRNAs), stem cell therapy, gasotransmitter donors (e.g., H_2_S), and non-pharmacological interventions (e.g., exercise training). Collectively, these strategies aim to suppress inflammation, attenuate remodeling, and preserve cardiac function.

## 8 Conclusion and prospect

NF-κB has emerged as a pivotal molecular integrator linking inflammation, cellular stress responses, metabolic dysregulation, and vascular remodeling in PAH. Increasing evidence indicates that persistent activation of NF-κB signaling contributes to disease progression through coordinated regulation of endothelial dysfunction, PASMC proliferation, inflammatory cell recruitment, extracellular matrix remodeling, EndMT, and RV maladaptation. Multiple pathogenic stimuli, including hypoxia, oxidative stress, endoplasmic reticulum stress, and innate immune activation, converge on NF-κB-centered signaling networks, including TLR4/NF-κB/HIF-1α, PERK/eIF2α/NF-κB, MAPK/ERK/NF-κB, and STAT3/NF-κB pathways. These findings position NF-κB not simply as an inflammatory mediator but as a central coordinator of the complex molecular interactions underlying PAH progression.

Despite substantial advances in understanding NF-κB biology in PAH, several important questions remain unresolved. First, the cellular and spatial heterogeneity of NF-κB activation within the pulmonary vascular microenvironment requires further investigation. Current evidence is largely derived from bulk tissue analysis or individual cell populations, which may overlook distinct NF-κB activation patterns among pulmonary endothelial cells, PASMCs, adventitial fibroblasts, macrophages, and adaptive immune cells. Future studies integrating single-cell RNA sequencing (scRNA-seq), single-cell chromatin accessibility profiling, spatial transcriptomics, and proteomic approaches may generate comprehensive maps of cell-specific NF-κB activity during different stages of PAH development. Such strategies may identify pathogenic cellular subsets that are particularly dependent on NF-κB signaling and provide a rationale for precision-targeted intervention.

Second, the complex interaction between NF-κB and other disease-driving pathways warrants deeper mechanistic exploration. Although current studies have demonstrated extensive crosstalk between NF-κB and HIF-1α-mediated hypoxic responses, STAT3-dependent inflammation, metabolic reprogramming, BMPR2 dysfunction, and non-coding RNA networks, the temporal sequence and regulatory hierarchy of these interactions remain unclear. The study on the loss of gene function provides important but incomplete evidence for the crucial role of NF-κB in the pathogenesis of PAH. Li et al. used mutant mice with a mutated IκBα driven by the Club (Clara) cell 10 promoter - whose lungs’ NF-κB activation was continuously blocked - and found that monocrotaline-induced PAH, right ventricular hypertrophy, endothelial cell apoptosis, and endothelial-to-mesenchymal transition were all inhibited (198). These results indicate that inhibiting NF-κB in the pulmonary region is sufficient to alleviate the key pathological features of PAH. However, such genetic research interpretations are subject to several limitations. Firstly, traditionally knocking out the NF-κB trans-activating subunit RelA/p65 or the upstream kinase IKKβ leads to embryonic lethality due to severe hepatocyte apoptosis, thus making it impossible to use conventional loss-of-function methods (199, 200). Although conditional knockout strategies have been developed, their application in PAH models is still limited, and the roles of NF-κB in different cell types have not been systematically elucidated. Secondly, most genetic studies are conducted in acute monocrotaline or hypoxia models rather than in genetic susceptible or chronic progressive PAH models, which limits their translational significance for human diseases (198). There is functional redundancy among the members of the NF-κB family (RelA, RelB, c-Rel, p50, and p52), making the interpretation of single-gene knockout phenotypes complex as compensatory mechanisms may mask the complete pathogenic contribution of NF-κB activation to disease occurrence (201, 202). These limitations suggest that although NF-κB is significantly activated and functionally relevant in PAH, current evidence is insufficient to prove that it is an indispensable driver of disease occurrence and progression, rather than merely a regulatory enhancer.

Third, therapeutic translation of NF-κB inhibition remains challenging because NF-κB is essential for physiological immune surveillance and tissue homeostasis. Therefore, future therapeutic strategies should move beyond nonspecific systemic inhibition toward selective modulation of disease-associated NF-κB signaling. Advances in nanotechnology, including nanoparticle-based drug delivery systems, may enable preferential delivery of NF-κB inhibitors or nucleic acid-based therapeutics to affected vascular cells while minimizing systemic toxicity. In addition, combination approaches targeting NF-κB together with complementary pathways, such as HIF-1α, STAT3, metabolic regulators, or epigenetic modifiers, may provide greater therapeutic efficacy than single-pathway inhibition.

Finally, identifying reliable biomarkers for NF-κB activation and treatment response represents an essential step toward clinical translation. Circulating inflammatory mediators, NF-κB-regulated microRNAs, long non-coding RNAs, extracellular vesicles, and tissue-specific molecular signatures may serve as potential biomarkers for patient stratification and therapeutic monitoring. Integrating molecular profiling with clinical phenotyping may facilitate individualized treatment strategies and identify patients most likely to benefit from NF-κB-targeted therapies.

In conclusion, NF-κB represents a promising therapeutic target that connects inflammatory signaling with pulmonary vascular remodeling and RV dysfunction in PAH (41). Future integration of single-cell technologies, multi-omics approaches, targeted drug delivery platforms, and biomarker-guided clinical studies will be essential to overcome current limitations and translate NF-κB-centered strategies into effective disease-modifying therapies for PAH (119, 195, 203).

## References

[1] Johnson S Sommer N Cox-Flaherty K Weissmann N Ventetuolo CE Maron BA . Pulmonary hypertension: a contemporary review.Am J Respir Crit Care Med. (2023) 208:528–48. doi: 10.1164/rccm.202302-0327so 37450768 PMC10492255

[2] Boucherat O Bonnet S Provencher S Potus F . Anti-remodeling therapies in pulmonary arterial hypertension.Trends Pharmacol Sci. (2025) 46:674–91. doi: 10.1016/j.tips.2025.05.004 40541519

[3] Galiè N McLaughlin VV Rubin LJ Simonneau G . An overview of the 6th world symposium on pulmonary hypertension.Eur Respir J. (2019) 53:50–5. doi: 10.1183/13993003.02148-2018 PMC6351332 30552088

[4] Cober ND McCourt E Soares Godoy R Deng Y Schlosser K Safaie Qamsari E . Mapping disease-specific vascular cell populations responsible for obliterative arterial remodelling during the development of pulmonary arterial hypertension.Cardiovasc Res. (2025) 121:2095–112. doi: 10.1093/cvr/cvaf146 40875786 PMC12560791

[5] Ruopp NF Cockrill BA . Diagnosis and treatment of pulmonary arterial hypertension: a review.Jama. (2022) 327:1379–91. doi: 10.1001/jama.2022.4402 35412560

[6] Farber HW Miller DP Poms AD Badesch DB Frost AE Muros-Le Rouzic E . Five-year outcomes of patients enrolled in the REVEAL Registry.Chest. (2015) 148:1043–54. doi: 10.1378/chest.15-0300 26066077

[7] Yang Y Zeng Z Yang Q Wang H Zhang H Yan W . The challenge in burden of pulmonary arterial hypertension: a perspective from the Global Burden of Disease Study.MedComm (2020). (2025) 6:e70175. doi: 10.1002/mco2.70175 40276646 PMC12019876

[8] Qiu JY Qiu LH Huang SS Qiu BC Liu C Ding D . Pulmonary arterial hypertension: a long-standing and nonnegligible burden over three decades.Sci Bull (Beijing). (2025) 70:1766–9. doi: 10.1016/j.scib.2025.04.014 40253296

[9] Liu Z Mo L Cao W Wang K Gong H Li C . Global, regional, and national trends in pulmonary arterial hypertension burden, 1990-2021: findings from the global burden of disease study 2021.Front Public Health. (2025) 13:1516365. doi: 10.3389/fpubh.2025.1516365 40510578 PMC12158705

[10] Cao Y Wang R He X Ding Y Chang Y Yang R . Targeted delivery of BMPR2 mRNA attenuates pulmonary arterial hypertension by reversing pulmonary vascular remodeling.Acta Pharm Sin B. (2025) 15:5416–30. doi: 10.1016/j.apsb.2025.07.004 41132833 PMC12541623

[11] Sitbon O Boucly A Weatherald J Antigny F Guignabert C Jevnikar M . Drugs targeting novel pathways in pulmonary arterial hypertension.Eur Respir J. (2025) 66:121–7. doi: 10.1183/13993003.01830-2024 40341051

[12] Hoeper MM Preston IR Gomberg-Maitland M Waxman AB Shi Y Lin J . Pericardial effusion in sotatercept phase 3 trials: insights from STELLAR and ZENITH.Eur Respir J. (2025) 66:127–33. doi: 10.1183/13993003.00768-2025 40774811 PMC12461899

[13] Preston IR Badesch D Ghofrani HA Gibbs JSR Gomberg-Maitland M Hoeper MM . A long-term follow-up study of sotatercept for treatment of pulmonary arterial hypertension: interim results of SOTERIA.Eur Respir J. (2025) 66. doi: 10.1183/13993003.01435-2024 39978862 PMC12287605

[14] Humbert M McLaughlin VV Badesch DB Ghofrani HA Gibbs JSR Gomberg-Maitland M . Sotatercept in patients with pulmonary arterial hypertension at high risk for death.N Engl J Med. (2025) 392:1987–2000. doi: 10.1056/nejmoa2415160 40167274

[15] McLaughlin VV Hoeper MM Badesch DB Ghofrani HA Gibbs JSR Gomberg-Maitland M . Sotatercept for pulmonary arterial hypertension within the first year after diagnosis.N Engl J Med. (2025) 393:1599–611. doi: 10.1056/nejmoa2508170 41025556

[16] Montani D McLaughlin VV Gibbs JSR Gomberg-Maitland M Hoeper MM Preston IR . Consistent safety and efficacy of sotatercept for pulmonary arterial hypertension in BMPR2 mutation carriers and noncarriers: a planned analysis of a phase II, double-blind, placebo-controlled clinical trial (PULSAR).Am J Respir Crit Care Med. (2025) 211:1028–37. doi: 10.1164/rccm.202409-1698oc 40035659

[17] Hoeper MM Badesch DB Ghofrani HA Gibbs JSR Gomberg-Maitland M McLaughlin VV . Phase 3 trial of sotatercept for treatment of pulmonary arterial hypertension.N Engl J Med. (2023) 388:1478–90. doi: 10.1056/nejmoa2213558 36877098

[18] Correale M Mercurio V Bevere EML Pezzuto B Tricarico L Attanasio U . Pathophysiology of pulmonary arterial hypertension: focus on vascular endothelium as a potential therapeutic target.Int J Mol Sci. (2025) 26:156–60. doi: 10.3390/ijms26199631 41096896 PMC12525453

[19] Guo Q Jin Y Chen X Ye X Shen X Lin M . NF-κB in biology and targeted therapy: new insights and translational implications.Signal Transduct Target Ther. (2024) 9:53. doi: 10.1038/s41392-024-01757-9 38433280 PMC10910037

[20] Mao H Zhao X Sun SC . NF-κB in inflammation and cancer. Cell Mol Immunol. (2025) 22:811–39. doi: 10.1038/s41423-025-01310-w PMC12310982 40562870

[21] Bassères DS Baldwin AS . Nuclear factor-kappaB and inhibitor of kappaB kinase pathways in oncogenic initiation and progression.Oncogene. (2006) 25:6817–30. doi: 10.1038/sj.onc.1209942 17072330

[22] Yamazaki S . The nuclear NF-κB regulator IκBζ: updates on its molecular functions and pathophysiological roles.Cells. (2024) 13:165–70. doi: 10.3390/cells13171467 39273036 PMC11393961

[23] Hong Y Fu Y Long Q . Post-translational governance of NF-κB in cancer immunity: mechanisms and therapeutic horizons.Front Immunol. (2025) 16:1627084. doi: 10.3389/fimmu.2025.1627084 41035656 PMC12479462

[24] Liu Y Wang J Zhang X . An update on the multifaceted role of NF-kappaB in endometriosis.Int J Biol Sci. (2022) 18:4400–13. doi: 10.7150/ijbs.72707 35864971 PMC9295070

[25] Mitchell S Tsui R Tan ZC Pack A Hoffmann A . The NF-κB multidimer system model: a knowledge base to explore diverse biological contexts.Sci Signal. (2023) 16:eabo2838. doi: 10.1126/scisignal.abo2838 36917644 PMC10195159

[26] Poma P . NF-κB and disease.Int J Mol Sci. (2020) 21:198–203. doi: 10.3390/ijms21239181

[27] Aqdas M Sung MH . NF-κB dynamics in the language of immune cells.Trends Immunol. (2023) 44:32–43. doi: 10.1016/j.it.2022.11.005 36473794 PMC9811507

[28] Pfisterer M Dreute J Schmitz ML . Insights from human NF-κB knockouts.EMBO Rep. (2025) 26:3491–505. doi: 10.1038/s44319-025-00500-x 40533538 PMC12287409

[29] Sadati S Khalaji A Bonyad A Khoshdooz S Hosseini Kolbadi KS Bahrami A . NF-κB and apoptosis: colorectal cancer progression and novel strategies for treatment.Eur J Med Res. (2025) 30:616. doi: 10.1186/s40001-025-02734-w 40660346 PMC12261797

[30] Liu D Zhang H Zhang Y Xiao L Wang J Liao S . Interaction between stromal cells and tumor cells promotes GCB-DLBCL cell survival via the CD40/RANK-KDM6B-NF-κB axis.Mol Ther. (2025) 33:3407–22. doi: 10.1016/j.ymthe.2025.03.025 40119515 PMC12265957

[31] Dhaka N Misra S Sahoo S Mukherjee SP . Biophysical characterization of RelA-p52 NF-κB dimer-a link between the canonical and the non-canonical NF-κB pathway.Protein Sci. (2024) 33:e5184. doi: 10.1002/pro.5184 39412374 PMC11481211

[32] Tando T Ishizaka A Watanabe H Ito T Iida S Haraguchi T . Requiem protein links RelB/p52 and the Brm-type SWI/SNF complex in a noncanonical NF-kappaB pathway.J Biol Chem. (2010) 285:21951–60. doi: 10.1074/jbc.m109.087783 20460684 PMC2903353

[33] Sun SC . The non-canonical NF-κB pathway in immunity and inflammation.Nat Rev Immunol. (2017) 17:545–58. doi: 10.1038/nri.2017.52 28580957 PMC5753586

[34] Cildir G Low KC Tergaonkar V . Noncanonical NF-κB signaling in health and disease.Trends Mol Med. (2016) 22:414–29. doi: 10.1016/j.molmed.2016.03.002 27068135

[35] Kucharzewska P Maracle CX Jeucken KCM van Hamburg JP Israelsson E Furber M . NIK-IKK complex interaction controls NF-κB-dependent inflammatory activation of endothelium in response to LTβR ligation.J Cell Sci. (2019) 132:249–50. doi: 10.1242/jcs.225615 30837284

[36] Kew RR Penzo M Habiel DM Marcu KB . The IKKα-dependent NF-κB p52/RelB noncanonical pathway is essential to sustain a CXCL12 autocrine loop in cells migrating in response to HMGB1.J Immunol. (2012) 188:2380–6. doi: 10.4049/jimmunol.1102454 22287708 PMC3288724

[37] Penzo M Habiel DM Ramadass M Kew RR Marcu KB . Cell migration to CXCL12 requires simultaneous IKKα and IKKβ-dependent NF-κB signaling.Biochim Biophys Acta. (2014) 1843:1796–804. doi: 10.1016/j.bbamcr.2014.04.011 PMC4096130 24747690

[38] Aravamudhan A Dieffenbach PB Choi KM Link PA Meridew JA Haak AJ . Non-canonical IKB kinases regulate YAP/TAZ and pathological vascular remodeling behaviors in pulmonary artery smooth muscle cells.Physiol Rep. (2024) 12:e15999. doi: 10.14814/phy2.15999 38610069 PMC11014870

[39] Trares K Ackermann J Koch I . The canonical and non-canonical NF-κB pathways and their crosstalk: a comparative study based on Petri nets.Biosystems. (2022) 211:104564. doi: 10.1016/j.biosystems.2021.104564 34688841

[40] Madge LA Kluger MS Orange JS May MJ . Lymphotoxin-alpha 1 beta 2 and LIGHT induce classical and noncanonical NF-kappa B-dependent proinflammatory gene expression in vascular endothelial cells.J Immunol. (2008) 180:3467–77. doi: 10.4049/jimmunol.180.5.3467 PMC2596750 18292573

[41] Si M Hu Y Jin Z Mao Y Kang L . NF-κB and pulmonary hypertension: advances in mechanistic research and therapeutic applications.Am Heart J Plus. (2025) 60:100669. doi: 10.1016/j.ahjo.2025.100669 41323511 PMC12664054

[42] Maimaitiaili N Zeng Y Ju P Zhakeer G E G Yao H . NLRC3 deficiency promotes hypoxia-induced pulmonary hypertension development via IKK/NF-κB p65/HIF-1α pathway.Exp Cell Res. (2023) 431:113755. doi: 10.1016/j.yexcr.2023.113755 37586455

[43] Mahomed AS Burke-Gaffney A Moledina S Toe QK Shao D Quinlan GJ . SOX17-silenced HPAECs upregulate NF-κB-induced CXCL10 and CXCL11: implications for lymphocyte chemotaxis in SOX17-PAH.Sci Rep. (2025) 15:34262. doi: 10.1038/s41598-025-16418-2 41034322 PMC12488963

[44] Zhao Z Sang M Li Q Zhang H Luo Z Zhang Y . Targeted FTO knockout in endothelial cells boosts adhesion and lowers inflammatory infiltration to alleviate pulmonary arterial hypertension.Biochem Biophys Res Commun. (2025) 749:151339. doi: 10.1016/j.bbrc.2025.151339 39869947

[45] Garcia-Hernandez ML Rangel-Moreno J Xu Q Jeong Y Bhattacharya S Misra R . TNF drives aberrant BMP signaling to induce endothelial and mesenchymal dysregulation in pulmonary hypertension.JCI Insight. (2025) 10:395–402. doi: 10.1172/jci.insight.174456 40569693 PMC12288976

[46] Li D Chen Y Wang Y Liu J Chai L Zhang Q . NAMPT mediates PDGF-induced pulmonary arterial smooth muscle cell proliferation by TLR4/NF-κB/PLK4 signaling pathway.Eur J Pharmacol. (2023) 961:176151. doi: 10.1016/j.ejphar.2023.176151 37914064

[47] Li Y Liu S Zhang Y Gao Q Sun W Fu L . Histone demethylase JARID1B regulates proliferation and migration of pulmonary arterial smooth muscle cells in mice with chronic hypoxia-induced pulmonary hypertension via nuclear factor-kappa B (NFkB).Cardiovasc Pathol. (2018) 37:8–14. doi: 10.1016/j.carpath.2018.07.004 30172777

[48] Yang D Sun C Zhang J Lin S Zhao L Wang L . Proliferation of vascular smooth muscle cells under inflammation is regulated by NF-κB p65/microRNA-17/RB pathway activation.Int J Mol Med. (2018) 41:43–50. doi: 10.3892/ijmm.2017.3212 29115381 PMC5746293

[49] Li Y Yang L Dong L Yang ZW Zhang J Zhang SL . Crosstalk between the Akt/mTORC1 and NF-κB signaling pathways promotes hypoxia-induced pulmonary hypertension by increasing DPP4 expression in PASMCs.Acta Pharmacol Sin. (2019) 40:1322–33. doi: 10.1038/s41401-019-0272-2 31316183 PMC6786428

[50] Mei L Zheng YM Song T Yadav VR Joseph LC Truong L . Rieske iron-sulfur protein induces FKBP12. 6/RyR2 complex remodeling and subsequent pulmonary hypertension through NF-κB/cyclin D1 pathway.Nat Commun. (2020) 11:3527. doi: 10.1038/s41467-020-17314-1 32669538 PMC7363799

[51] Liu J Li Q Zeng C Wang Y Hu Q Wang H . Role of myelin and lymphocyte protein in regulating pulmonary artery smooth muscle cell proliferation and apoptosis in pulmonary hypertension.Nan Fang Yi Ke Da Xue Xue Bao. (2022) 42:1572–7. doi: 10.12122/j.issn.1673-4254.2022.10.19 PMC9637499 36329594

[52] Chen M Ding Z Zhang F Shen H Zhu L Yang H . A20 attenuates hypoxia-induced pulmonary arterial hypertension by inhibiting NF-κB activation and pulmonary artery smooth muscle cell proliferation.Exp Cell Res. (2020) 390:111982. doi: 10.1016/j.yexcr.2020.111982 32234376

[53] Zhu N Zhao X Xiang Y Ye S Huang J Hu W . Thymoquinone attenuates monocrotaline-induced pulmonary artery hypertension via inhibiting pulmonary arterial remodeling in rats.Int J Cardiol. (2016) 221:587–96. doi: 10.1016/j.ijcard.2016.06.192 27420584

[54] Thenappan T Ormiston ML Ryan JJ Archer SL . Pulmonary arterial hypertension: pathogenesis and clinical management.Bmj. (2018) 360:j5492. doi: 10.1136/bmj.j5492 29540357 PMC6889979

[55] Xiao Y Cai Z Bai M Wu X Wang W Liu R . Harnessing NLRX1: a new frontier in mitigating inflammation in pulmonary hypertension.Respir Res. (2025) 26:311. doi: 10.1186/s12931-025-03364-w 41204207 PMC12595859

[56] Zhang H Li M Hu CJ Stenmark KR . Fibroblasts in pulmonary hypertension: roles and molecular mechanisms.Cells. (2024) 13:587–9. doi: 10.3390/cells13110914 38891046 PMC11171669

[57] Prasad RR Kumar S Zhang H Li M Hu CJ Riddle S . An intracellular complement system drives metabolic and proinflammatory reprogramming of vascular fibroblasts in pulmonary hypertension.JCI Insight. (2025) 10:593–618. doi: 10.1172/jci.insight.184141 39946184 PMC11949053

[58] Thenappan T Chan SY Weir EK . Role of extracellular matrix in the pathogenesis of pulmonary arterial hypertension.Am J Physiol Heart Circ Physiol. (2018) 315:H1322–h1331. doi: 10.1152/ajpheart.00136.2018 30141981 PMC6297810

[59] Pienkos S Gallego N Condon DF Cruz-Utrilla A Ochoa N Nevado J . Novel TNIP2 and TRAF2 variants are implicated in the pathogenesis of pulmonary arterial hypertension.Front Med (Lausanne). (2021) 8:625763. doi: 10.3389/fmed.2021.625763 33996849 PMC8119639

[60] Davies RJ Holmes AM Deighton J Long L Yang X Barker L . BMP type II receptor deficiency confers resistance to growth inhibition by TGF-β in pulmonary artery smooth muscle cells: role of proinflammatory cytokines.Am J Physiol Lung Cell Mol Physiol. (2012) 302:L604–15. doi: 10.1152/ajplung.00309.2011 22227206 PMC3311534

[61] Tian W Jiang X Sung YK Shuffle E Wu TH Kao PN . Phenotypically silent bone morphogenetic protein receptor 2 mutations predispose rats to inflammation-induced pulmonary arterial hypertension by enhancing the risk for neointimal transformation.Circulation. (2019) 140:1409–25. doi: 10.1161/circulationaha.119.040629 31462075 PMC6803052

[62] Tian XT Peng ZYF Wu YS Cao YY Li XC Li Y . Loss of type 2 bone morphogenetic protein receptor activates NOD-like receptor family protein 3/Gasdermin E-mediated pyroptosis in pulmonary arterial hypertension.J Am Heart Assoc. (2025) 14:e034726. doi: 10.1161/jaha.124.034726 39846318 PMC12074700

[63] Deng L Cao C Cai Z Wang Z Leng B Chen Z . STING contributes to pulmonary hypertension by targeting IFN and BMPR2 signaling through regulating of F2RL3.Am J Respir Cell Mol Biol. (2024) 71:356–71. doi: 10.1165/rcmb.2023-0308oc 38864771

[64] Li L Kim IK Chiasson V Chatterjee P Gupta S . NF-κB mediated miR-130a modulation in lung microvascular cell remodeling: implication in pulmonary hypertension.Exp Cell Res. (2017) 359:235–42. doi: 10.1016/j.yexcr.2017.07.024 28755990

[65] Toro V Mougin M Brossat C Jambon-Barbara C Hlavaty A Guay CA . Breast cancer reveals latent BMPR2-related susceptibility to pulmonary hypertension.Circulation. (2026) 153:516–33. doi: 10.1161/circulationaha.125.079067 41603037 PMC12908636

[66] Kim HJ Kim H Lee JH Hwangbo C . Toll-like receptor 4 (TLR4): new insight immune and aging.Immun Ageing. (2023) 20:67. doi: 10.1186/s12979-023-00383-3 38001481 PMC10668412

[67] Zhang Y Liang X Bao X Xiao W Chen G . Toll-like receptor 4 (TLR4) inhibitors: current research and prospective.Eur J Med Chem. (2022) 235:114291. doi: 10.1016/j.ejmech.2022.114291 35307617

[68] Bolourani S Brenner M Wang P . The interplay of DAMPs, TLR4, and proinflammatory cytokines in pulmonary fibrosis.J Mol Med (Berl). (2021) 99:1373–84. doi: 10.1007/s00109-021-02113-y 34258628 PMC8277227

[69] Ren W Zhao L Sun Y Wang X Shi X . HMGB1 and toll-like receptors: potential therapeutic targets in autoimmune diseases.Mol Med. (2023) 29:117. doi: 10.1186/s10020-023-00717-3 37667233 PMC10478470

[70] Luo Z Tian M Yang G Tan Q Chen Y Li G . Hypoxia signaling in human health and diseases: implications and prospects for therapeutics.Signal Transduct Target Ther. (2022) 7:218. doi: 10.1038/s41392-022-01080-1 35798726 PMC9261907

[71] Zhang J Zhang W Yang Z Fan B Wang C Tian Z . Cinnamaldehyde alleviates pulmonary hypertension by affecting vascular remodeling through the TLR4/NF-kB/HIF-1a pathway.Clin Exp Hypertens. (2025) 47:2486829. doi: 10.1080/10641963.2025.2486829 40171680

[72] Wang EL Zhang JJ Luo FM Fu MY Li D Peng J . Cerebellin-2 promotes endothelial-mesenchymal transition in hypoxic pulmonary hypertension rats by activating NF-κB/HIF-1α/Twist1 pathway.Life Sci. (2023) 328:121879. doi: 10.1016/j.lfs.2023.121879 37355224

[73] Feng M An Y Qin Q Fong IH Zhang K Wang F . Sphk1/S1P pathway promotes blood-brain barrier breakdown after intracerebral hemorrhage through inducing Nlrp3-mediated endothelial cell pyroptosis.Cell Death Dis. (2024) 15:926. doi: 10.1038/s41419-024-07310-4 39715736 PMC11666774

[74] Bu Y Wu H Deng R Wang Y . Therapeutic potential of SphK1 inhibitors based on abnormal expression of SphK1 in inflammatory immune related-diseases.Front Pharmacol. (2021) 12:733387. doi: 10.3389/fphar.2021.733387 34737701 PMC8560647

[75] Mirhadi E Roufogalis BD Banach M Barati M Sahebkar A . Resveratrol: mechanistic and therapeutic perspectives in pulmonary arterial hypertension.Pharmacol Res. (2021) 163:105287. doi: 10.1016/j.phrs.2020.105287 33157235

[76] Ren G Jiao P Yan Y Ma X Qin G . Baicalin exerts a protective effect in diabetic nephropathy by repressing inflammation and oxidative stress through the SphK1/S1P/NF-κB signaling pathway.Diabetes Metab Syndr Obes. (2023) 16:1193–205. doi: 10.2147/dmso.s407177 37131503 PMC10149099

[77] Youssef NS Elzaitony AS Abdel Baky NA . Diacerein attenuate LPS-induced acute lung injury via inhibiting ER stress and apoptosis: impact on the crosstalk between SphK1/S1P, TLR4/NFκB/STAT3, and NLRP3/IL-1β signaling pathways.Life Sci. (2022) 308:120915. doi: 10.1016/j.lfs.2022.120915 36055546

[78] Shi W Zhai C Feng W Wang J Zhu Y Li S . Resveratrol inhibits monocrotaline-induced pulmonary arterial remodeling by suppression of SphK1-mediated NF-κB activation.Life Sci. (2018) 210:140–9. doi: 10.1016/j.lfs.2018.08.071 30179628

[79] Pyne NJ Pyne S . Sphingosine kinase 1: a potential therapeutic target in pulmonary arterial hypertension? Trends Mol Med. (2017) 23:786–98. doi: 10.1016/j.molmed.2017.07.001 28789830

[80] Yeager ME Reddy MB Nguyen CM Colvin KL Ivy DD Stenmark KR . Activation of the unfolded protein response is associated with pulmonary hypertension.Pulm Circ. (2012) 2:229–40. doi: 10.4103/2045-8932.97613 22837864 PMC3401877

[81] Sun Y Liu S Chen C Yang S Pei G Lin M . The mechanism of programmed death and endoplasmic reticulum stress in pulmonary hypertension.Cell Death Discov. (2023) 9:78. doi: 10.1038/s41420-023-01373-6 36841823 PMC9968278

[82] Yang Q Lai B Xie H Deng M Li J Yang Y . Identification of differentially expressed ER stress-related genes and their association with pulmonary arterial hypertension.Respir Res. (2024) 25:220. doi: 10.1186/s12931-024-02849-4 38789967 PMC11127292

[83] Price LC Wort SJ Perros F Dorfmüller P Huertas A Montani D . Inflammation in pulmonary arterial hypertension.Chest. (2012) 141:210–21. doi: 10.1378/chest.11-0793 22215829

[84] Chen A Liu J Zhu J Wang X Xu Z Cui Z . FGF21 attenuates hypoxia-induced dysfunction and apoptosis in HPAECs through alleviating endoplasmic reticulum stress.Int J Mol Med. (2018) 42:1684–94. doi: 10.3892/ijmm.2018.3705 29845288

[85] Wang JL Liu H Jing ZC Zhao F Zhou R . 18β-Glycyrrhetinic acid ameliorates endoplasmic reticulum stress-induced inflammation in pulmonary arterial hypertension through PERK/eIF2α/NF-κB signaling.Chin J Physiol. (2022) 65:187–98. doi: 10.4103/0304-4920.354801 36073567

[86] Fawzy MA Maher SA Bakkar SM El-Rehany MA Fathy M . Pantoprazole attenuates MAPK (ERK1/2, JNK, p38)-NF-κB and apoptosis signaling pathways after renal ischemia/reperfusion injury in rats.Int J Mol Sci. (2021) 22:931–5. doi: 10.3390/ijms221910669 34639009 PMC8508698

[87] Fang JY Richardson BC . The MAPK signalling pathways and colorectal cancer.Lancet Oncol. (2005) 6:322–7. doi: 10.1016/s1470-2045(05)70168-6 15863380

[88] Lu X Bijli KM Ramirez A Murphy TC Kleinhenz J Hart CM . Hypoxia downregulates PPARγ via an ERK1/2-NF-κB-Nox4-dependent mechanism in human pulmonary artery smooth muscle cells.Free Radic Biol Med. (2013) 63:151–60. doi: 10.1016/j.freeradbiomed.2013.05.013 23684777 PMC3729594

[89] Hosokawa S Haraguchi G Sasaki A Arai H Muto S Itai A . Pathophysiological roles of nuclear factor kappaB (NF-kB) in pulmonary arterial hypertension: effects of synthetic selective NF-kB inhibitor IMD-0354.Cardiovasc Res. (2013) 99:35–43. doi: 10.1093/cvr/cvt105 23631839

[90] Bijli KM Kleinhenz JM Murphy TC Kang BY Adesina SE Sutliff RL . Peroxisome proliferator-activated receptor gamma depletion stimulates Nox4 expression and human pulmonary artery smooth muscle cell proliferation.Free Radic Biol Med. (2015) 80:111–20. doi: 10.1016/j.freeradbiomed.2014.12.019 25557278 PMC4355175

[91] Ricard N Scott RP Booth CJ Velazquez H Cilfone NA Baylon JL . Endothelial ERK1/2 signaling maintains integrity of the quiescent endothelium.J Exp Med. (2019) 216:1874–90. doi: 10.1084/jem.20182151 31196980 PMC6683988

[92] Mussa A Carli D Giorgio E Villar AM Cardaropoli S Carbonara C . MEK inhibition in a newborn with RAF1-associated Noonan syndrome ameliorates hypertrophic cardiomyopathy but is insufficient to revert pulmonary vascular disease.Genes (Basel). (2021) 13:991–4. doi: 10.3390/genes13010006 35052347 PMC8774485

[93] Chaudhry A Carthan KA Kang BY Calvert J Sutliff RL Hart CM . PPARγ attenuates hypoxia-induced hypertrophic transcriptional pathways in the heart.Pulm Circ. (2017) 7:98–107. doi: 10.1086/689749 28680569 PMC5448534

[94] Wang C Qin L Zhang X Liu Y . Robinin attenuates cardiac hypertrophy in pulmonary heart disease by modulating SIRT1/NF-κB signaling and inhibiting oxidative stress and the NLRP3 inflammasome.Hum Exp Toxicol. (2025) 44:9603271251361892. doi: 10.1177/09603271251361892 40679082

[95] Shimauchi T Boucherat O Yokokawa T Grobs Y Wu W Orcholski M . PARP1-PKM2 axis mediates right ventricular failure associated with pulmonary arterial hypertension.JACC Basic Transl Sci. (2022) 7:384–403. doi: 10.1016/j.jacbts.2022.01.005 35540097 PMC9079853

[96] Imoto K Okada M Yamawaki H . Periostin mediates right ventricular failure through induction of inducible nitric oxide synthase expression in right ventricular fibroblasts from monocrotaline-induced pulmonary arterial hypertensive rats.Int J Mol Sci. (2018) 20:1058–63. doi: 10.3390/ijms20010062 30586863 PMC6337160

[97] Novoyatleva T Janssen W Wietelmann A Schermuly RT Engel FB . TWEAK/Fn14 axis is a positive regulator of cardiac hypertrophy.Cytokine. (2013) 64:43–5. doi: 10.1016/j.cyto.2013.05.009 23764551

[98] Ishikawa T Abe K Takana-Ishikawa M Yoshida K Watanabe T Imakiire S . Chronic inhibition of toll-like receptor 9 ameliorates pulmonary hypertension in rats.J Am Heart Assoc. (2021) 10:e019247. doi: 10.1161/jaha.120.019247 33787285 PMC8174358

[99] Kang S Tanaka T Narazaki M Kishimoto T . Targeting interleukin-6 signaling in clinic.Immunity. (2019) 50:1007–23. doi: 10.1016/j.immuni.2019.03.026 30995492

[100] Wu O Wu Y Zhang X Liu W Zhang H Khederzadeh S . Causal effect of interleukin (IL)-6 on blood pressure and hypertension: a mendelian randomization study.Immunogenetics. (2024) 76:123–35. doi: 10.1007/s00251-024-01332-0 38427105

[101] Styczeń A Krysa M Mertowska P Grywalska E Urbanowicz T Krasiński M . The role of toll-like receptors and viral infections in the pathogenesis and progression of pulmonary arterial hypertension-a narrative review.Int J Mol Sci. (2025) 26:1098–100. doi: 10.3390/ijms262211143 PMC12653618 41303627

[102] Tomaszewski M Mertowska P Janczewska M Styczeń A Mertowski S Jonas K . In the search for biomarkers of pulmonary arterial hypertension, are cytokines IL-2, IL-4, IL-6, IL-10, and IFN-gamma the right indicators to use? Int J Mol Sci. (2023) 24:1104–6. doi: 10.3390/ijms241813694 37761997 PMC10530884

[103] Penumatsa KC Sharma Y Warburton RR Singhal A Toksoz D Bhedi CD . Lung-specific interleukin 6 mediated transglutaminase 2 activation and cardiopulmonary fibrogenesis.Front Immunol. (2024) 15:1371706. doi: 10.3389/fimmu.2024.1371706 38650935 PMC11033445

[104] Dendorfer U Oettgen P Libermann TA . Multiple regulatory elements in the interleukin-6 gene mediate induction by prostaglandins, cyclic AMP, and lipopolysaccharide.Mol Cell Biol. (1994) 14:4443–54. doi: 10.1128/mcb.14.7.4443-4454.1994 8007951 PMC358816

[105] Hirano T . IL-6 in inflammation, autoimmunity and cancer.Int Immunol. (2021) 33:127–48. doi: 10.1093/intimm/dxaa078 33337480 PMC7799025

[106] Atschekzei F Traidl S Carlens J Schütz K von Hardenberg S Elsayed A . JAK inhibitors to treat STAT3 gain-of-function: a single-center report and literature review.Front Immunol. (2024) 15:1400348. doi: 10.3389/fimmu.2024.1400348 39247195 PMC11377292

[107] Guanizo AC Fernando CD Garama DJ Gough DJ . STAT3: a multifaceted oncoprotein.Growth Factors. (2018) 36:1–14. doi: 10.1080/08977194.2018.1473393 29873274

[108] Ishibashi T Inagaki T Okazawa M Yamagishi A Ohta-Ogo K Asano R . IL-6/gp130 signaling in CD4(+) T cells drives the pathogenesis of pulmonary hypertension.Proc Natl Acad Sci USA. (2024) 121:e2315123121. doi: 10.1073/pnas.2315123121 38602915 PMC11032454

[109] Paulin R Meloche J Bonnet S . STAT3 signaling in pulmonary arterial hypertension.Jakstat. (2012) 1:223–33. doi: 10.4161/jkst.22366 24058777 PMC3670278

[110] Fu M Luo F Wang E Jiang Y Liu S Peng J . Magnolol attenuates right ventricular hypertrophy and fibrosis in hypoxia-induced pulmonary arterial hypertensive rats through inhibition of the JAK2/STAT3 signaling pathway.Front Pharmacol. (2021) 12:755077. doi: 10.3389/fphar.2021.755077 34764873 PMC8576411

[111] Peng SL . Interplay between the NF-kappaB and forkhead transcription factors.Cell Death Differ. (2005) 12:699–701. doi: 10.1038/sj.cdd.4401640 15861187

[112] Zouein FA Kurdi M Booz GW . Dancing rhinos in stilettos: the amazing saga of the genomic and nongenomic actions of STAT3 in the heart.Jakstat. (2013) 2:e24352. doi: 10.4161/jkst.24352 24069556 PMC3772108

[113] Karim A Garg R Saikia B Tiwari A Sahu S Malhotra M . Unraveling the unphosphorylated STAT3-unphosphorylated NF-κB pathway in loss of function STAT3 hyper IgE syndrome.Front Immunol. (2024) 15:1332817. doi: 10.3389/fimmu.2024.1332817 39229272 PMC11369709

[114] Koudstaal T van Hulst JAC Das T Neys SFH Merkus D Bergen IM . DNGR1-Cre-mediated deletion of Tnfaip3/A20 in conventional dendritic cells induces pulmonary hypertension in mice.Am J Respir Cell Mol Biol. (2020) 63:665–80. doi: 10.1165/rcmb.2019-0443OC 32755457

[115] Guignabert C . Dendritic cells in pulmonary hypertension: foot soldiers or hidden enemies? Am J Respir Cell Mol Biol. (2020) 63:551–2. doi: 10.1165/rcmb.2020-0330ed 32804536 PMC7605161

[116] van Uden D Koudstaal T van Hulst JAC Bergen IM Gootjes C Morrell NW . Central role of dendritic cells in pulmonary arterial hypertension in human and mice.Int J Mol Sci. (2021) 22:1154–7. doi: 10.3390/ijms22041756 33578743 PMC7916474

[117] Imakiire S Kimuro K Yoshida K Masaki K Izumi R Imabayashi M . Activated factor X inhibition ameliorates NF-κB-IL-6-mediated perivascular inflammation and pulmonary hypertension.Am J Physiol Lung Cell Mol Physiol. (2025) 329:L183–96. doi: 10.1152/ajplung.00303.2024 40514194

[118] Guo L Wang L Qin G Zhang J Peng J Li L . M-type pyruvate kinase 2 (PKM2) tetramerization alleviates the progression of right ventricle failure by regulating oxidative stress and mitochondrial dynamics.J Transl Med. (2023) 21:888. doi: 10.1186/s12967-023-04780-6 38062516 PMC10702013

[119] Hong J Arneson D Umar S Ruffenach G Cunningham CM Ahn IS . Single-cell study of two rat models of pulmonary arterial hypertension reveals connections to human pathobiology and drug repositioning.Am J Respir Crit Care Med. (2021) 203:1006–22. doi: 10.1164/rccm.202006-2169oc 33021809 PMC8048757

[120] Toshner M Church C Harbaum L Rhodes C Villar Moreschi SS Liley J . Mendelian randomisation and experimental medicine approaches to interleukin-6 as a drug target in pulmonary arterial hypertension.Eur Respir J. (2022) 59:1183–7. doi: 10.1183/13993003.02463-2020 34588193 PMC8907935

[121] Medrek S Melendres-Groves L . Evolving nonvasodilator treatment options for pulmonary arterial hypertension.Curr Opin Pulm Med. (2022) 28:361–8. doi: 10.1097/mcp.0000000000000887 35838352

[122] Becher C Groeneveld EJ Quarck R Neep B Pan X Szulcek R . BMP9 modulates IL-33 signaling to mitigate EndMT in pulmonary arterial hypertension.Hypertension. (2026) 83:e24916. doi: 10.1161/hypertensionaha.125.24916 41564150 PMC12822782

[123] Li X Wang F Liu N Liu Y Yu W Tang M . Lactate promotes endothelial-mesenchymal transition via mediating Twist1 lactylation in hypoxic pulmonary hypertension.Int J Mol Sci. (2026) 27:1204–11. doi: 10.3390/ijms27052255 41828479 PMC12984667

[124] Chen X Su J Liu H Qin Y Li M Xie P . Endothelial-to-mesenchymal transition mechanisms in vascular remodeling of pulmonary hypertension.Int J Mol Sci. (2026) 27:1204–11. doi: 10.3390/ijms27114951 42278476 PMC13257180

[125] Piera-Velazquez S Jimenez SA . Endothelial to mesenchymal transition: role in physiology and in the pathogenesis of human diseases.Physiol Rev. (2019) 99:1281–324. doi: 10.1152/physrev.00021.2018 30864875 PMC6734087

[126] Zhang Z Fang Z Ge J Li H . Endothelial-to-mesenchymal transition in cardiovascular diseases.Trends Mol Med. (2025) 31:992–1007. doi: 10.1016/j.molmed.2025.05.002 40467364

[127] Guo Z Li L Luo J Xue W Bian S Zhu Y . The Ets2 super-enhancer modulates endothelial-mesenchymal transition during cardiac ageing.Cardiovasc Res. (2026) 121:2892–908. doi: 10.1093/cvr/cvaf242 41253710

[128] Tian S Cai Z Sen P van Uden D van de Kamp E Thuillet R . Loss of lung microvascular endothelial Piezo2 expression impairs NO synthesis, induces EndMT, and is associated with pulmonary hypertension.Am J Physiol Heart Circ Physiol. (2022) 323:H958–74. doi: 10.1152/ajpheart.00220.2022 36149769

[129] Lee HW Adachi T Pak B Park S Hu X Choi W . BMPR1A promotes ID2-ZEB1 interaction to suppress excessive endothelial to mesenchymal transition.Cardiovasc Res. (2023) 119:813–25. doi: 10.1093/cvr/cvac159 36166408 PMC10409893

[130] Wu Y Cai C Xiang Y Zhao H Lv L Zeng C . Naringin ameliorates monocrotaline-induced pulmonary arterial hypertension through endothelial-to-mesenchymal transition inhibition.Front Pharmacol. (2021) 12:696135. doi: 10.3389/fphar.2021.696135 34335261 PMC8320371

[131] Li Y Lui KO Zhou B . Reassessing endothelial-to-mesenchymal transition in cardiovascular diseases.Nat Rev Cardiol. (2018) 15:445–56. doi: 10.1038/s41569-018-0023-y 29748594

[132] Good RB Gilbane AJ Trinder SL Denton CP Coghlan G Abraham DJ . Endothelial to mesenchymal transition contributes to endothelial dysfunction in pulmonary arterial hypertension.Am J Pathol. (2015) 185:1850–8. doi: 10.1016/j.ajpath.2015.03.019 25956031

[133] Zhu L Lama S Tu L Dusting GJ Wang JH Liu GS . TAK1 signaling is a potential therapeutic target for pathological angiogenesis.Angiogenesis. (2021) 24:453–70. doi: 10.1007/s10456-021-09787-5 33973075

[134] Ma M Dang Y Chang B Wang F Xu J Chen L . TAK1 is an essential kinase for STING trafficking.Mol Cell. (2023) 83:3885–3903.e5. doi: 10.1016/j.molcel.2023.09.009 37832545

[135] Morales-Cano D Izquierdo-García JL Barreira B Esquivel-Ruiz S Callejo M Pandolfi R . Impact of a TAK-1 inhibitor as a single or as an add-on therapy to riociguat on the metabolic reprograming and pulmonary hypertension in the SUGEN5416/hypoxia rat model. Front Pharmacol. (2023) 14:1021535. doi: 10.3389/fphar.2023.1021535 37063275 PMC10090662

[136] Yu M Wu X Wang J He M Han H Hu S . Paeoniflorin attenuates monocrotaline-induced pulmonary arterial hypertension in rats by suppressing TAK1-MAPK/NF-κB pathways. Int J Med Sci. (2022) 19:681–94. doi: 10.7150/ijms.69289 35582418 PMC9108400

[137] Zhang W Li X Li M He H Yang C Wang M . Empagliflozin inhibits neointimal hyperplasia through attenuating endothelial-to-mesenchymal transition via TAK-1/NF-κB pathway. Eur J Pharmacol. (2023) 954:175826. doi: 10.1016/j.ejphar.2023.175826 37321472

[138] Peltzer N Walczak H . Endothelial cell killing by TAK1 inhibition: a novel anti-angiogenic strategy in cancer therapy. Dev Cell. (2019) 48:127–8. doi: 10.1016/j.devcel.2019.01.011 30695692

[139] Nasim MT Ogo T Chowdhury HM Zhao L Chen CN Rhodes C . BMPR-II deficiency elicits pro-proliferative and anti-apoptotic responses through the activation of TGFβ-TAK1-MAPK pathways in PAH. Hum Mol Genet. (2012) 21:2548–58. doi: 10.1093/hmg/dds073 22388934

[140] Eckenstaler R Hauke M Benndorf RA . A current overview of RhoA, RhoB, and RhoC functions in vascular biology and pathology. Biochem Pharmacol. (2022) 206:115321. doi: 10.1016/j.bcp.2022.115321 36306821

[141] Oka M Homma N Taraseviciene-Stewart L Morris KG Kraskauskas D Burns N . Rho kinase-mediated vasoconstriction is important in severe occlusive pulmonary arterial hypertension in rats. Circ Res. (2007) 100:923–9. doi: 10.1007/978-1-60761-500-2_19 17332430

[142] Soh JEC Shimizu A Sato A Ogita H . Novel cardiovascular protective effects of RhoA signaling and its therapeutic implications.Biochem Pharmacol. (2023) 218:115899. doi: 10.1016/j.bcp.2023.115899 37907138

[143] Chen N Tu Y Liu DQ Zhang Y Tian YK Zhou YQ . Exploring the role of RhoA/ROCK signaling in pain: a narrative review.Aging Dis. (2025) 17:981–1001. doi: 10.14336/ad.2024.1539 40249935 PMC12834413

[144] Saleh MA Shaaban AA Talaat IM Elmougy A Adra S Ahmad F . RhoA/ROCK inhibition attenuates endothelin-1-induced glomerulopathy in the rats.Life Sci. (2023) 323:121687. doi: 10.1016/j.lfs.2023.121687 37030613

[145] Mori M Sakamoto A Kawakami R Guo L Slenders L Verdezoto Mosquera J . CD163(+) macrophages induce endothelial-to-mesenchymal transition in atheroma. Circ Res. (2024) 135:e4–e23. doi: 10.1161/circresaha.123.324082 38860377

[146] Kim HJ Kim JG Moon MY Park SH Park JB . IκB kinase γ/nuclear factor-κB-essential modulator (IKKγ/NEMO) facilitates RhoA GTPase activation, which, in turn, activates Rho-associated KINASE (ROCK) to phosphorylate IKKβ in response to transforming growth factor (TGF)-β1.J Biol Chem. (2014) 289:1429–40. doi: 10.1074/jbc.M113.520130 PMC3894326 24240172

[147] Wang Y Duo D Yan Y He R Wu X . Magnesium lithospermate B ameliorates hypobaric hypoxia-induced pulmonary arterial hypertension by inhibiting endothelial-to-mesenchymal transition and its potential targets.BioMed Pharmacother. (2020) 130:110560. doi: 10.1016/j.biopha.2020.110560 34321157

[148] Hindmarch CCT Potus F Al-Qazazi R Ott BP Nichols WC Rauh MJ . Tet methylcytosine dioxygenase 2 (TET2) mutation drives a global hypermethylation signature in patients with pulmonary arterial hypertension (PAH): correlation with altered gene expression relevant to a common T cell phenotype.Compr Physiol. (2025) 15:e70011. doi: 10.1002/cph4.70011 40274312 PMC12021535

[149] Mumby S Gambaryan N Meng C Perros F Humbert M Wort SJ . Bromodomain and extra-terminal protein mimic JQ1 decreases inflammation in human vascular endothelial cells: implications for pulmonary arterial hypertension.Respirology. (2017) 22:157–64. doi: 10.1111/resp.12872 27539364 PMC5215513

[150] Hudson J Farkas L . Epigenetic regulation of endothelial dysfunction and inflammation in pulmonary arterial hypertension.Int J Mol Sci. (2021) 22:1458–60. doi: 10.3390/ijms222212098 34829978 PMC8617605

[151] Lopes-Pacheco M . Extracellular vesicles: multimodal tools for diagnosis, prognosis, and therapy in respiratory diseases.Expert Rev Mol Med. (2025) 27:e38. doi: 10.1017/erm.2025.10025 41115837 PMC12671918

[152] Polini B Ghelardoni S Capone G Monti E Chiellini G Lionetti V . Circulating microRNAs in pulmonary arterial hypertension: biomarkers for diagnosis, prognostic stratification, and treatment.Geroscience. (2026) 48:2243–66. doi: 10.1007/s11357-026-02095-0 41526595 PMC12972461

[153] Lago-Docampo M Iglesias-López A Vilariño C Baloira A Barberá JA Blanco I . A circulating microRNA signature for the diagnosis of pulmonary arterial hypertension and functional characterization of candidate miR-3168.Sci Rep. (2026) 16:1468–72. doi: 10.1038/s41598-026-42550-8 41786847 PMC13076637

[154] Fan J Fan X Guang H Shan X Tian Q Zhang F . Upregulation of miR-335-3p by NF-κB transcriptional regulation contributes to the induction of pulmonary arterial hypertension via APJ during hypoxia.Int J Biol Sci. (2020) 16:515–28. doi: 10.7150/ijbs.34517 32015687 PMC6990898

[155] Yan J Wang A Cao J Chen L . Apelin/APJ system: an emerging therapeutic target for respiratory diseases.Cell Mol Life Sci. (2020) 77:2919–30. doi: 10.1007/s00018-020-03461-7 32128601 PMC11105096

[156] Yan Y Tian LY Jia Q Han Y Tian Y Chen HN . MiR-130a-3p regulates FUNDC1-mediated mitophagy by targeting GJA1 in myocardial ischemia/reperfusion injury.Cell Death Discov. (2023) 9:77. doi: 10.21203/rs.3.rs-1740501/v2 36841811 PMC9968299

[157] Bertero T Cottrill K Krauszman A Lu Y Annis S Hale A . The microRNA-130/301 family controls vasoconstriction in pulmonary hypertension.J Biol Chem. (2015) 290:2069–85. doi: 10.1074/jbc.m114.617845 25505270 PMC4303661

[158] Han JJ . LncRNAs: the missing link to senescence nuclear architecture.Trends Biochem Sci. (2023) 48:618–28. doi: 10.1016/j.tibs.2023.03.007 37069045

[159] Tai YY Yu Q Tang Y Sun W Kelly NJ Okawa S . Allele-specific control of rodent and human lncRNA KMT2E-AS1 promotes hypoxic endothelial pathology in pulmonary hypertension.Sci Transl Med. (2024) 16:eadd2029. doi: 10.1126/scitranslmed.add2029 38198571 PMC10947529

[160] Han Y Ali MK Dua K Spiekerkoetter E Mao Y . Role of long non-coding RNAs in pulmonary arterial hypertension.Cells. (2021) 10:1503–6. doi: 10.3390/cells10081892 34440661 PMC8394897

[161] Wołowiec Ł Mędlewska M Osiak J Wołowiec A Grześk E Jaśniak A . MicroRNA and lncRNA as the future of pulmonary arterial hypertension treatment.Int J Mol Sci. (2023) 24:1506–8. doi: 10.3390/ijms24119735 PMC10253568 37298685

[162] Chen H Li Q Liang J Jin M Lu A . LncRNA CPS1-IT1 serves as anti-oncogenic role in glioma. BioMed Pharmacother. (2019) 118:109277. doi: 10.1016/j.biopha.2019.109277 31545272

[163] Zhang Z Li Z Wang Y Wei L Chen H . Overexpressed long noncoding RNA CPS1-IT alleviates pulmonary arterial hypertension in obstructive sleep apnea by reducing interleukin-1β expression via HIF1 transcriptional activity.J Cell Physiol. (2019) 234:19715–27. doi: 10.1002/jcp.28571 30982984

[164] Zhang W Yuan W Song J Wang S Gu X . Lncrna CPS1-IT1 suppresses cell proliferation, invasion and metastasis in colorectal cancer.Cell Physiol Biochem. (2017) 44:567–80. doi: 10.1159/000485091 29145177

[165] Bredel M Espinosa L Kim H Scholtens DM McElroy JP Rajbhandari R . Haploinsufficiency of NFKBIA reshapes the epigenome antipodal to the IDH mutation and imparts disease fate in diffuse gliomas.Cell Rep Med. (2023) 4:101082. doi: 10.1016/j.xcrm.2023.101082 37343523 PMC10314122

[166] Archer SL Marsboom G Kim GH Zhang HJ Toth PT Svensson EC . Epigenetic attenuation of mitochondrial superoxide dismutase 2 in pulmonary arterial hypertension: a basis for excessive cell proliferation and a new therapeutic target.Circulation. (2010) 121:2661–71. doi: 10.1161/circulationaha.109.916098 20529999 PMC2914302

[167] Emon IM Al-Qazazi R Rauh MJ Archer SL . The role of clonal hematopoiesis of indeterminant potential and DNA (cytosine-5)-methyltransferase dysregulation in pulmonary arterial hypertension and other cardiovascular diseases.Cells. (2023) 12:1554–6. doi: 10.3390/cells12212528 37947606 PMC10650407

[168] Potus F Pauciulo MW Cook EK Zhu N Hsieh A Welch CL . Novel mutations and decreased expression of the epigenetic regulator TET2 in pulmonary arterial hypertension.Circulation. (2020) 141:1986–2000. doi: 10.1161/circulationaha.119.044320 32192357 PMC7299806

[169] Qiu JY Huang SS Liu C Ding D Xu YH Mao YM . Genetic evidence for the causal effect of clonal hematopoiesis on pulmonary arterial hypertension.BMC Cardiovasc Disord. (2025) 25:38. doi: 10.1186/s12872-025-04475-4 39849426 PMC11755826

[170] Chelladurai P Boucherat O Stenmark K Kracht M Seeger W Bauer UM . Targeting histone acetylation in pulmonary hypertension and right ventricular hypertrophy.Br J Pharmacol. (2021) 178:54–71. doi: 10.1111/bph.14932 31749139

[171] Diao W Liu G Shi C Jiang Y Li H Meng J . Evaluating the effect of Circ-Sirt1 on the expression of SIRT1 and its role in pathology of pulmonary hypertension.Cell Transplant. (2022) 31:9636897221081479. doi: 10.1177/09636897221081479 35225027 PMC9114726

[172] Yeung F Hoberg JE Ramsey CS Keller MD Jones DR Frye RA . Modulation of NF-kappaB-dependent transcription and cell survival by the SIRT1 deacetylase.EMBO J. (2004) 23:2369–80. doi: 10.1038/sj.emboj.7600244 15152190 PMC423286

[173] Santos-Barriopedro I Bosch-Presegué L Marazuela-Duque A de la Torre C Colomer C Vazquez BN . SIRT6-dependent cysteine monoubiquitination in the PRE-SET domain of Suv39h1 regulates the NF-κB pathway.Nat Commun. (2018) 9:101. doi: 10.1038/s41467-017-02586-x 29317652 PMC5760577

[174] Mumby S Perros F Grynblat J Manaud G Papi A Casolari P . Differential responses of pulmonary vascular cells from PAH patients and controls to TNFα and the effect of the BET inhibitor JQ1.Respir Res. (2023) 24:193. doi: 10.1186/s12931-023-02499-y 37516840 PMC10386603

[175] Meloche J Potus F Vaillancourt M Bourgeois A Johnson I Deschamps L . Bromodomain-containing protein 4: the epigenetic origin of pulmonary arterial hypertension.Circ Res. (2015) 117:525–35. doi: 10.1161/circresaha.115.307004 26224795

[176] Dave J Jagana V Janostiak R Bisserier M . Unraveling the epigenetic landscape of pulmonary arterial hypertension: implications for personalized medicine development.J Transl Med. (2023) 21:477. doi: 10.1186/s12967-023-04339-5 37461108 PMC10353122

[177] Aljubran SA Cox Jr R Parthasarathy PT Ramanathan GK Rajanbabu V Bao H . Enhancer of zeste homolog 2 induces pulmonary artery smooth muscle cell proliferation. PloS One. (2012) 7:e37712. doi: 10.1371/journal.pone.0037712 22662197 PMC3360676

[178] Wang Y Huang XX Leng D Li JF Liang Y Jiang T . Effect of EZH2 on pulmonary artery smooth muscle cell migration in pulmonary hypertension.Mol Med Rep. (2021) 23:1628–32. doi: 10.3892/mmr.2020.11768 33313943 PMC7751464

[179] Ea CK Baltimore D . Regulation of NF-kappaB activity through lysine monomethylation of p65.Proc Natl Acad Sci USA. (2009) 106:18972–7. doi: 10.1073/pnas.0910439106 19864627 PMC2770010

[180] Chen J Chen Y Chen W Tan Y Wang T Xie Y . Set7 regulates ferroptosis in pulmonary arterial hypertension endothelial cells through the DPP4/NOX4 axis mediated by H3K4me1 modification.Cell Signal. (2026) 139:112352. doi: 10.1016/j.cellsig.2026.112352 41500376

[181] Chen F Wang H Yan J Lai J Cai S Yuan L . Grape seed proanthocyanidin reverses pulmonary vascular remodeling in monocrotaline-induced pulmonary arterial hypertension by down-regulating HSP70.BioMed Pharmacother. (2018) 101:123–8. doi: 10.1016/j.biopha.2018.02.037 29482057

[182] Wu Y Cai C Yang L Xiang Y Zhao H Zeng C . Inhibitory effects of formononetin on the monocrotaline-induced pulmonary arterial hypertension in rats.Mol Med Rep. (2020) 21:1192–200. doi: 10.3892/mmr.2020.10911 31922224 PMC7003019

[183] Sánchez-Gloria JL Martínez-Olivares CE Rojas-Morales P Hernández-Pando R Carbó R Rubio-Gayosso I . Anti-inflammatory effect of allicin associated with fibrosis in pulmonary arterial hypertension.Int J Mol Sci. (2021) 22:1806–14. doi: 10.3390/ijms22168600 34445305 PMC8395330

[184] Yang JM Zhou R Zhang M Tan HR Yu JQ . Betaine attenuates monocrotaline-induced pulmonary arterial hypertension in rats via inhibiting inflammatory response.Molecules. (2018) 23:1806–14. doi: 10.3390/molecules23061274 29861433 PMC6100216

[185] Li M Ying M Gu S Zhou Z Zhao R . Matrine alleviates hypoxia-induced inflammation and pulmonary vascular remodelling via RPS5/NF-κB signalling pathway.J Biochem Mol Toxicol. (2024) 38:e23583. doi: 10.1002/jbt.23583 37986032

[186] Wang GK Li SH Zhao ZM Liu SX Zhang GX Yang F . Inhibition of heat shock protein 90 improves pulmonary arteriole remodeling in pulmonary arterial hypertension.Oncotarget. (2016) 7:54263–73. doi: 10.18632/oncotarget.10855 27472464 PMC5342340

[187] Lee JH Park BK Oh KS Yi KY Lim CJ Seo HW . A urotensin II receptor antagonist, KR36676, decreases vascular remodeling and inflammation in experimental pulmonary hypertension.Int Immunopharmacol. (2016) 40:196–202. doi: 10.1016/j.intimp.2016.09.002 27611861

[188] Uwe S . Anti-inflammatory interventions of NF-kappaB signaling: potential applications and risks.Biochem Pharmacol. (2008) 75:1567–79. doi: 10.1016/j.bcp.2007.10.027 18070616

[189] Häcker H Karin M . Regulation and function of IKK and IKK-related kinases. Sci STKE. (2006) 2006:re13. doi: 10.1126/stke.3572006re13 17047224

[190] Begalli F Bennett J Capece D Verzella D D'Andrea D Tornatore L . Unlocking the NF-κB conundrum: embracing complexity to achieve specificity.Biomedicines. (2017) 5:1841–4. doi: 10.3390/biomedicines5030050 28829404 PMC5618308

[191] Kimura S Egashira K Chen L Nakano K Iwata E Miyagawa M . Nanoparticle-mediated delivery of nuclear factor kappaB decoy into lungs ameliorates monocrotaline-induced pulmonary arterial hypertension.Hypertension. (2009) 53:877–83. doi: 10.1161/hypertensionaha.108.121418 19307469

[192] Zhang D Wang X Chen S Chen S Yu W Liu X . Endogenous hydrogen sulfide sulfhydrates IKKβ at cysteine 179 to control pulmonary artery endothelial cell inflammation.Clin Sci (Lond). (2019) 133:2045–59. doi: 10.1042/cs20190514 31654061

[193] Nogueira-Ferreira R Moreira-Gonçalves D Silva AF Duarte JA Leite-Moreira A Ferreira R . Exercise preconditioning prevents MCT-induced right ventricle remodeling through the regulation of TNF superfamily cytokines.Int J Cardiol. (2016) 203:858–66. doi: 10.1016/j.ijcard.2015.11.066 26599752

[194] Zoulikha M Zou J Yang P Wu J Wu W Hao K . Cocrystal pleomorphism-inspired drug nanoassembly for pulmonary-endothelium targeting and pulmonary hypertension treatment.Acta Pharm Sin B. (2025) 15:557–70. doi: 10.1016/j.apsb.2024.11.008 40041921 PMC11873612

[195] Park JS Choi YH Min JY Lee J Shim G . Fundamental and targeted approaches in pulmonary arterial hypertension treatment.Pharmaceutics. (2025) 17:1878–82. doi: 10.3390/pharmaceutics17020224 40006591 PMC11859843

[196] Kanaya T Miyagawa S Kawamura T Sakai Y Masada K Nawa N . Innovative therapeutic strategy using prostaglandin I(2) agonist (ONO1301) combined with nano drug delivery system for pulmonary arterial hypertension.Sci Rep. (2021) 11:7292. doi: 10.1038/s41598-021-86781-3 33790393 PMC8012709

[197] Schupp JC Adams TS Cosme Jr C Raredon MSB Yuan Y Omote N . Integrated single-cell atlas of endothelial cells of the human lung.Circulation. (2021) 144:286–302. doi: 10.1161/circulationaha.120.052318 34030460 PMC8300155

[198] Li L Wei C Kim IK Janssen-Heininger Y Gupta S . Inhibition of nuclear factor-κB in the lungs prevents monocrotaline-induced pulmonary hypertension in mice.Hypertension. (2014) 63:1260–9. doi: 10.1161/hypertensionaha.114.03220 24614212

[199] Beg AA Sha WC Bronson RT Ghosh S Baltimore D . Embryonic lethality and liver degeneration in mice lacking the RelA component of NF-kappa B.Nature. (1995) 376:167–70. doi: 10.1038/376167a0 7603567

[200] Li Q Van Antwerp D Mercurio F Lee KF Verma IM . Severe liver degeneration in mice lacking the IkappaB kinase 2 gene.Science. (1999) 284:321–5. doi: 10.1126/science.284.5412.321 10195897

[201] Pasparakis M Luedde T Schmidt-Supprian M . Dissection of the NF-kappaB signalling cascade in transgenic and knockout mice.Cell Death Differ. (2006) 13:861–72. doi: 10.1038/sj.cdd.4401870 16470223

[202] Hoffmann A Leung TH Baltimore D . Genetic analysis of NF-kappaB/Rel transcription factors defines functional specificities.EMBO J. (2003) 22:5530–9. doi: 10.1093/emboj/cdg534 14532125 PMC213788

[203] Khassafi F Chelladurai P Valasarajan C Nayakanti SR Martineau S Sommer N . Transcriptional profiling unveils molecular subgroups of adaptive and maladaptive right ventricular remodeling in pulmonary hypertension.Nat Cardiovasc Res. (2023) 2:917–36. doi: 10.1038/s44161-023-00338-3 39196250 PMC11358157

